# The Lipid A 1-Phosphatase, LpxE, Functionally Connects Multiple Layers of Bacterial Envelope Biogenesis

**DOI:** 10.1128/mBio.00886-19

**Published:** 2019-06-18

**Authors:** Jinshi Zhao, Jinsu An, Dohyeon Hwang, Qinglin Wu, Su Wang, Robert A. Gillespie, Eun Gyeong Yang, Ziqiang Guan, Pei Zhou, Hak Suk Chung

**Affiliations:** a Department of Biochemistry, Duke University Medical Center, Durham, North Carolina, USA; b Center for Theragnosis, Biomedical Research Institute, Korea Institute of Science and Technology, Seoul, Republic of Korea; c Division of Bio-Medical Science & Technology, KIST School, Korea University of Science and Technology, Seoul, Republic of Korea; University of Georgia; Fred Hutchinson Cancer Research Center

**Keywords:** bacterial cell envelope biogenesis, lipid A 1-phosphate phosphatase, phosphatidylglycerol phosphate phosphatase, type 2 phosphatidic acid phosphatase (PAP2) superfamily, undecaprenyl pyrophosphate phosphatase

## Abstract

Although distinct lipid phosphatases are thought to be required for processing lipid A (component of the outer leaflet of the outer membrane), glycerophospholipid (component of the inner membrane and the inner leaflet of the outer membrane), and undecaprenyl pyrophosphate (C_55_-PP; precursors of peptidoglycan and O antigens of lipopolysaccharide) in Gram-negative bacteria, we report that the lipid A 1-phosphatases, LpxEs, functionally connect multiple layers of cell envelope biogenesis in Gram-negative bacteria. We found that Aquifex aeolicus LpxE structurally resembles YodM in Bacillus subtilis, a phosphatase for phosphatidylglycerol phosphate (PGP) with a weak *in vitro* activity on C_55_-PP, and rescues Escherichia coli deficient in PGP and C_55_-PP phosphatase activities; deletion of *lpxE* in Francisella novicida reduces the MIC value of bacitracin, indicating a significant contribution of LpxE to the native bacterial C_55_-PP phosphatase activity. Suppression of plasmid-borne *lpxE* in F. novicida deficient in chromosomally encoded C_55_-PP phosphatase activities results in cell enlargement, loss of O-antigen repeats of lipopolysaccharide, and ultimately cell death. These discoveries implicate LpxE as the first example of a multifunctional regulatory enzyme that orchestrates lipid A modification, O-antigen production, and peptidoglycan biogenesis to remodel multiple layers of the Gram-negative bacterial envelope.

## Abstract (precis)

Dephosphorylation of the lipid A 1-phosphate by LpxE in Gram-negative bacteria plays important roles in antibiotic resistance, bacterial virulence, and modulation of the host immune system. Our results demonstrate that in addition to removing the 1-phosphate from lipid A, LpxEs also dephosphorylate undecaprenyl pyrophosphate, an important metabolite for the synthesis of the essential envelope components, peptidoglycan and O-antigen. Therefore, LpxEs participate in multiple layers of biogenesis of the Gram-negative bacterial envelope and increase antibiotic resistance. This discovery marks an important step toward understanding the regulation and biogenesis of the Gram-negative bacterial envelope.

## INTRODUCTION

The Gram-negative bacterial cell envelope consists of three essential molecular architectures—the inner membrane, the peptidoglycan layer, and the outer membrane—that together protect bacteria against mechanical stress, maintain cell shape, and shield these microorganisms from the damage of detergents and antibiotics. These architectures are formed by distinct molecules, with phospholipids constituting the inner membrane and inner leaflet of the outer membrane, peptide-conjugated carbohydrates constituting the peptidoglycan layer, and lipopolysaccharides (LPS) anchoring at the outer leaflet of the outer membrane through the hydrophobic lipid A moiety. As peptidoglycan, phospholipids, and LPS are synthesized through distinct pathways, how Gram-negative bacteria orchestrate the biogenesis and remodeling across three layers of the cell envelope for optimal bacterial growth and virulence remains incompletely understood.

As the major lipid species coating the outer surface of Gram-negative bacteria, lipid A is the predominant signaling molecule that is detected by the mammalian Toll-like receptor 4 (TLR4)/myeloid differentiation factor 2 (MD-2) innate immune receptor (1) and caspase-4/-5/-11 (2) to trigger the host innate immune response to bacterial infection. With few exceptions, Gram-negative bacteria constitutively synthesize the 1,4′-bisphosphorylated tetra-acyl-lipid A intermediate, 3-deoxy-d-manno-oct-2-ulosonic acid (Kdo)-linked lipid IV_A_ (Kdo_2_-lipid IV_A_), via the action of seven conserved enzymes in the Raetz pathway (3) (see Fig. S1A in the supplemental material), which are essential to nearly all Gram-negative bacteria and are attractive targets for novel antibiotics (4–6). Gram-negative bacteria additionally harbor modification enzymes that further process the Kdo_2_-lipid IV_A_ intermediate to generate unique lipid A molecules in each bacterial species to adapt to environmental changes and evade the host immune response (7). For example, the lipid A 1-phosphate is a key determinant for lipid A recognition by the mammalian TLR4/MD-2 innate immune receptor (8). Removal of the lipid A 1-phosphate by the membrane-embedded phosphatase LpxE strongly protects bacteria against host cationic peptides and the last-resort antibiotic colistin (9), significantly dampens the host innate immune response, and dramatically increases colonization and survival of Helicobacter pylori in the gastric mucosa (10).

10.1128/mBio.00886-19.1 FIG S1 Lipid A biosynthesis and modification. (A) Schematic illustration of Kdo_2_-lipid A biosynthesis and modification. The lipid A 1-phosphatase LpxE is in pink. (B) Lipid A structure of A. pyrophilus. Download FIG S1, TIF file, 2.8 MB. Copyright © 2019 Zhao et al. 2019 Zhao et al. This content is distributed under the terms of the Creative Commons Attribution 4.0 International license.

In order to gain molecular insights into the structure and function of the lipid A 1-phosphatase LpxE, we identified the previously uncharacterized gene *aq_1706* from Aquifex aeolicus as the gene for the thermophilic LpxE enzyme (LpxE_AA_). Our structural analysis of LpxE_AA_ shows distinct features between LpxE_AA_ and Escherichia coli PgpB (PgpB_EC_) enzymes but reveals a surprising structural similarity to YodM, a phosphatase of phosphatidylglycerol phosphate (PGP) in the Gram-positive bacterium Bacillus subtilis with a weak *in vitro* activity on undecaprenyl pyrophosphate (C_55_-PP). Consistent with our structural analysis, we found that LpxE_AA_ possesses substantial *in vitro* activities toward Kdo_2_-lipid A/lipid IV_A_, C_55_-PP, and PGP and complements E. coli strains deficient in C_55_-PP phosphatase and PGP phosphatase activities. In addition to the LpxE enzyme from A. aeolicus, distant LpxE orthologs from *Francisella*, *Helicobacter*, and *Rhizobium* also complement E. coli strains deficient in the C_55_-PP phosphatase activity, supporting the notion that the multifunctional lipid phosphatase activity is a general feature of LpxE enzymes. Significantly, deletion of the native *lpxE* gene sensitizes Francisella novicida to bacitracin, an antibiotic that sequesters C_55_-PP to disrupt peptidoglycan synthesis; furthermore, suppression of plasmid-encoded *lpxE* in the F. novicida strain deficient in the endogenous C_55_-PP phosphatase activity results in noticeable changes in cell morphology, profound reduction of O-antigen repeats in LPS, and loss of cell viability. Taken together, these observations reveal a previously unappreciated contribution of LpxE to peptidoglycan biogenesis and LPS O-antigen modification beyond its well-recognized role as the lipid A 1-phosphatase to orchestrate the remodeling of multiple layers of the Gram-negative bacterial envelope to respond to environmental changes, evade host immune surveillance, and promote bacterial viability and virulence.

## RESULTS

### A distant ortholog of LpxE_FN_ in A. aeolicus.

LpxE is a member of the lipid phosphatase/phosphotransferase (LPT) family, a well-distributed family of lipid-processing enzymes also known as the integral transmembrane branch of the type II phosphatidic acid phosphatase (PAP2) superfamily (11, 12). This family is characterized by a conserved tripartite active site motif of KX_6_RP---PSGH---SRX_5_HX_3_D and activity independent of Mg^2+^ or other cations (13). The LPT family includes enzymes responsible for processing several types of lipids in Gram-negative bacteria, including the membrane-embedded PgpB, which dephosphorylates PGP and C_55_-PP (14) (Fig. S2). Even though PgpB and LpxE are both members of the LPT family, they have been reported to have distinct substrate specificities: PgpB is unable to utilize lipid A as a substrate (15), whereas purified LpxE from Rhizobium leguminosarum (LpxE_RL_) utilizes PGP ∼1,000 times less efficiently than lipid A species as a substrate *in vitro* (16).

10.1128/mBio.00886-19.2 FIG S2 Lipid phosphatases. Reactions of PGP and C_55_-PP phosphatases are shown in panels A and B, respectively. Download FIG S2, TIF file, 2.8 MB. Copyright © 2019 Zhao et al. 2019 Zhao et al. This content is distributed under the terms of the Creative Commons Attribution 4.0 International license.

In order to gain a molecular understanding of the LpxE structure and function, we searched for a thermophilic LpxE enzyme from *Aquificae* to facilitate structural analysis. The lipid A of Aquifex pyrophilus LPS contains d-galacturonic acid in place of phosphates at the 1- and 4′-positions (17) (Fig. S1B). As the 1,4′-bisphosphorylated lipid IV_A_ is a common lipid A intermediate before further modification (18, 19) and as *Aquificae* has the conserved biosynthetic enzymes to make 1,4′-bisphosphorylated lipid IV_A_, incorporation of the d-galacturonic acid moiety requires the removal of 1-phosphate from lipid A, indicating the presence of the lipid A 1-phosphatase activity in *Aquificae.* Such a rationale led us to search for the gene responsible for the lipid A 1-phosphatase activity in A. aeolicus VF5, as no lipid A 1-phosphatase has been reported in any *Aquifex* species.

**FIG 1:**
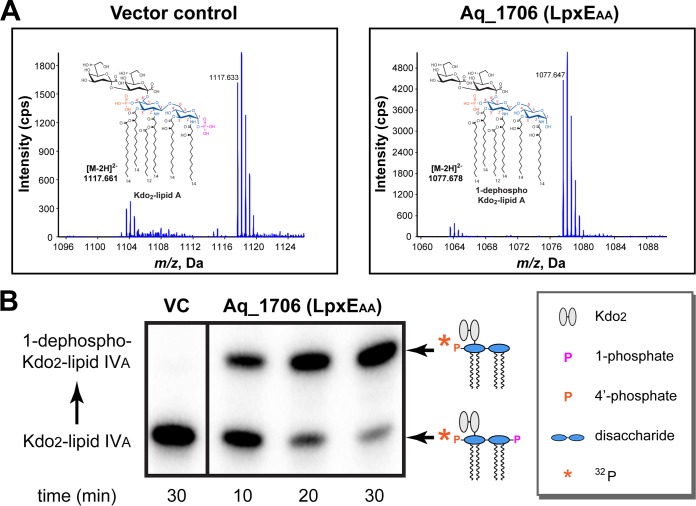
Characterization of Aq_1706 from A. aeolicus as the lipid A 1-phosphatase LpxE_AA_. (A) Mass spectrometry analysis of lipid A species in the heptose-deficient E. coli strain WBB06 (left) and the WBB06 strain overexpressing LpxE_AA_ (right). (B) ^32^P-autoradiographic TLC-based Kdo_2_-lipid IV_A_ 1-dephosphorylation assay of the membrane extract of the C41(DE3) strain overexpressing LpxE_AA_.

A Position-Specific Iterated Basic Local Alignment Search Tool (PSI-BLAST) (20) search revealed a distant ortholog of F. novicida LpxE (LpxE_FN_) (15), Aq_1706 (E value, 0.81; sequence identity, 13.84%), in the genome of A. aeolicus VF5. Aq_1706 shares little sequence identity with other LpxE enzymes (sequence identities of Aq_1706 with LpxE of *Helicobacter* and *Rhizobium* are 16.58% and 14.57%, respectively), except for the well-conserved tripartite active-site motif of KX_6_RP---PSGH---SRX_5_HX_3_D (Fig. S3). In order to determine if *aq_1706* encodes the lipid A 1-phosphatase activity *in vivo*, we overexpressed Aq_1706 in the heptose transferase-deficient E. coli strain WBB06, which produces Kdo_2_-lipid A instead of full-length LPS, to facilitate mass spectrometry analysis of lipid A modifications (21). Since E. coli does not encode LpxE activity, mass spectrometry analysis of the extracted lipids showed normal lipid A containing 1-phosphate with an *m/z* of 1,117.633 for the [M-2H]^2−^ ion species (calculated *m/z*, 1,117.661 for the exact mass of 2,237.336 of Kdo_2_-lipid A) from E. coli cells expressing a control vector; in contrast, overexpression of Aq_1706 in E. coli led to the disappearance of the intact lipid A species and significant accumulation of lipid A molecules lacking the 1-phosphate group, with an *m/z* of 1,077.647 for the [M-2H]^2−^ ion species (calculated *m/z*, 1,077.678 for the exact mass of 2,157.370 of 1-dephospho Kdo_2_-lipid A), consistent with the anticipated lipid A 1-phosphatase activity (Fig. 1A). In order to verify that the loss of phosphate occurred at the 1-position, but not at the 4′-position, we further tested the ability of LpxE to dephosphorylate 4′-^32^P-labeled Kdo_2_-lipid IV_A_, which was previously shown to be an efficient substrate for LpxE enzymes with specific activity comparable to that for the substrate Kdo_2_-lipid A (16). We found that treatment of Kdo_2_-[4′-^32^P] lipid IV_A_ with membrane extracts from E. coli overexpressing Aq_1706, but not those carrying a control vector, resulted in time-dependent reduction of the Kdo_2_-lipid IV_A_ band and accumulation of an upper-shifted band on the thin-layer chromatography (TLC) plate (Fig. 1B), reflecting the removal of 1-phosphate but retention of the ^32^P-labeled 4′-phosphate group. Taken together, these observations verify *aq_1706* in A. aeolicus as the gene that encodes the thermophilic lipid A 1-phosphatase LpxE (LpxE_AA_).

10.1128/mBio.00886-19.3 FIG S3 Sequence alignment of LpxE orthologs. Alignment was achieved by Clustal Omega (F. Sievers, A. Wilm, D. Dineen, T. J. Gibson, et al., Mol Syst Biol 7:539, 2011) with manual adjustment. Conserved residues in the signature motifs of the PAP2 family of enzymes are labeled. Download FIG S3, TIF file, 2.9 MB. Copyright © 2019 Zhao et al. 2019 Zhao et al. This content is distributed under the terms of the Creative Commons Attribution 4.0 International license.

### Structural analysis of LpxE_AA_ reveals a striking similarity to YodM in B. subtilis.

**FIG 2:**
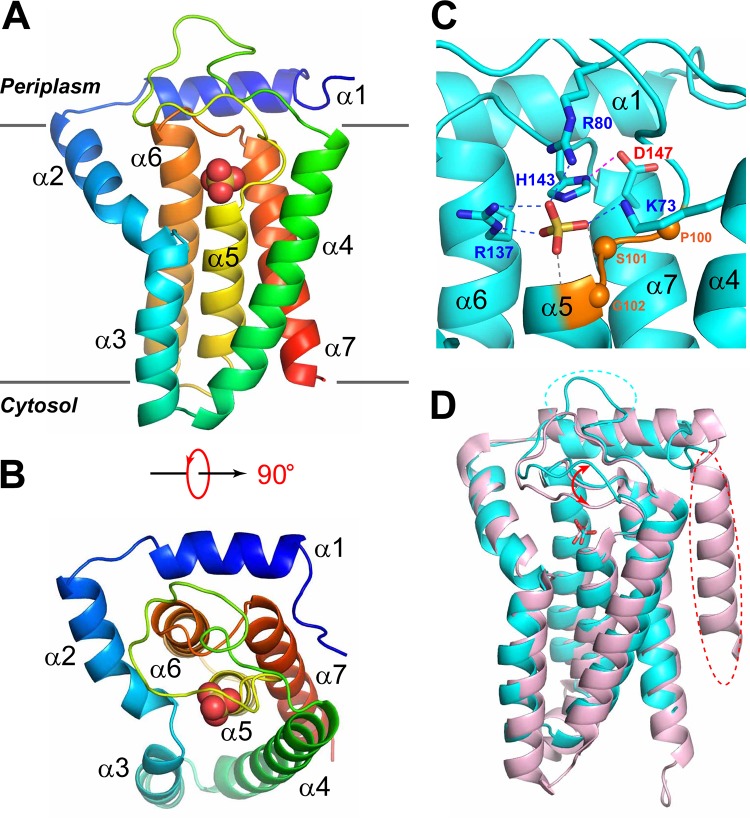
Crystal structure of LpxE_AA_. (A) Ribbon representation of LpxE_AA_, with blue to red colors corresponding to the N to C termini. The sulfate molecule is shown in the space-filling model. Individual helices and membrane locations are labeled. (B) Top view of LpxE_AA_. (C) The active site of LpxE_AA_. The sulfate molecule is shown in the stick model. Side chains of H143 and D147 from the RX_5_HX_3_D motif and conserved residues coordinating the sulfate molecule, including K73 and R80 from the KX_6_RP motif and R137 of the RX_5_HX_3_D motif, are shown in the stick model. Hydrogen bonds are shown by dashed lines. The sulfate group is additionally stabilized by the interaction with the electrical dipole of helix α5 (indicated by gray hydrogen bonds). The conserved PSG motif is colored in coral, with Cα atoms shown in spheres. (D) Superimposition of LpxE_AA_ (cyan) with YodM_BS_ (PDB code 5JKI; pink), revealing striking structural similarities. The major differences between the two structures are highlighted, with dashed circles indicating missing structural features (helix or loop) and arrows indicating conformational discrepancy.

After verifying the lipid A 1-phosphatase activity of LpxE_AA_, we cloned and purified LpxE_AA_. Consistent with the TMHMM analysis (http://www.cbs.dtu.dk/services/TMHMM/), high-yield expression of LpxE_AA_ was achieved in a maltose-binding protein (MBP) fusion construct containing an N-terminal PelB secretion signal (22), suggesting that the N terminus of LpxE_AA_ is located at the periplasmic side of the inner membrane. The crystal structure of LpxE_AA_ containing an I63M mutation was determined at 2.38 Å (Fig. 2A; statistics shown in Table S1). The selenomethionine substitution of the nonconserved I63 residue (I63M) was designed to enhance the selenium single anomalous dispersion (Se-SAD) signal for *de novo* phasing. The overall structure of LpxE_AA_ contains seven α-helices, including an N-terminal amphiphilic helix lying at the periplasmic surface of the inner membrane and five tightly packed transmembrane helices (α3 to α7). Apart from α2, which originates from the periplasmic surface and penetrates halfway across the inner membrane at an ∼45° angle and immediately connects to transmembrane helix α3, the remaining helices are oriented largely in parallel or antiparallel with each other and perpendicularly to the membrane plane. Looking from the periplasmic surface, helix 5 (α5) is located at the center, which is surrounded by α2, α3, α4, α7, and α6 in a counterclockwise fashion (Fig. 2B).

10.1128/mBio.00886-19.7 TABLE S1 Data collection and refinement statistics of LpxE_AA_. Download Table S1, DOCX file, 0.01 MB. Copyright © 2019 Zhao et al. 2019 Zhao et al. This content is distributed under the terms of the Creative Commons Attribution 4.0 International license.

The active site of LpxE is located at the periplasmic surface of the inner membrane and is defined by conserved motifs specific to the PAP2 enzymes (K^73^X_6_ R^80^P---R^137^X_5_ H^143^X_3_ D^147^) located at the C-terminal end of α4, the α4-α5 loop, α6, the α6-α7 loop, and the N terminus of α7 (Fig. 2C). Fortuitously, a sulfate molecule is found in the active site, which is a structural analog of the 1-phosphate group of lipid A. The sulfate group is extensively recognized by K73 and R80 of the K^73^X_6_R^80^P motif and R137 of the R^137^X_5_H^143^X_3_D^147^ motif. The catalytically important H143 is located 3.3 Å away from the sulfur atom of the sulfate group, ready to carry out inline attack to remove the phosphate group of the lipid substrate. D147, the last residue of the R^137^X_5_H^143^X_3_D^147^ motif, forms a hydrogen bond with H143. Although the corresponding aspartate residue is found in most LpxE enzymes (Fig. S3), it is absent in the LpxE ortholog from H. pylori (LpxE_HP_), suggesting that it is not absolutely required for catalysis. The first three residues of the PSGH motif are conserved in LpxE_AA_, with the central serine residue (S101) serving as a helix cap to stabilize helix α5, but the histidine residue is replaced with an aspartate residue in LpxE_AA_ (Fig. 2C).

The LpxE_AA_ structure shows noticeable conformational discrepancy with the previously reported structures of PgpB_EC_ (PDB codes 4PX7 and 5JWY) (23, 24), another PAP2 family enzyme, with overall backbone root mean square deviations (RMSDs) of ∼4.5 Å (Fig. S4); surprisingly, LpxE_AA_ is structurally similar to the recently reported YodM in B. subtilis (PDB code 5JKI) (25), a PGP phosphatase with a weak *in vitro* activity on C_55_-PP, with an overall backbone RMSD of 1.2 Å (Fig. 2D). The major differences of these two enzymes are the absence of an N-terminal transmembrane helix in LpxE_AA_ in comparison with YodM, a longer α4-α5 loop in LpxE_AA_, and a significant conformational variation of the α4-α5 loop surrounding the active site.

10.1128/mBio.00886-19.4 FIG S4 Superimposition of LpxE_AA_ with PgpB_EC_ structures**_._** RMSDs of ∼4.5 Å were observed between the coordinates of LpxE_AA_ and PgpB_EC_ (PDB codes 4PX7 [J. Fan, D. Jiang, Y. Zhao, J. Liu, et al., Proc Natl Acad Sci U S A 111:7636–7640, 2014] and 5JWY [S. L. Tong, Y. B. Lin, S. Lu, M. T. Wang, et al., J Biol Chem 291:18342–18352, 2016]). LpxE_AA_ and PgpB_EC_ are shown in Cα traces, with LpxE_AA_ colored in cyan and PgpB_EC_ in gray. Download FIG S4, TIF file, 2.8 MB. Copyright © 2019 Zhao et al. 2019 Zhao et al. This content is distributed under the terms of the Creative Commons Attribution 4.0 International license.

### LpxE_AA_ is a trifunctional lipid phosphatase *in vitro* and functionally complements E. coli mutants deficient in C_55_-PP or PGP phosphatase activities.

Surprised by the structural similarity between LpxE_AA_ and YodM_BS_, we asked whether LpxE_AA_ could function as a C_55_-PP and PGP phosphatase. To address this question, we compared the specific activities of purified LpxE_AA_ toward Kdo_2_-lipid A, PGP, and C_55_-PP using the malachite green assay to detect the release of inorganic phosphate. As expected, LpxE_AA_ efficiently catalyzed the hydrolysis of 1-phosphate from Kdo_2_-lipid A, with a specific activity of 2.04 ± 0.46 μmol/mg/min. Moreover, LpxE_AA_ catalyzed C_55_-PP more efficiently than it catalyzed Kdo_2_-lipid A, with a specific activity of 3.58 ± 0.47 μmol/mg/min—a value that is ∼1.8-fold higher than that toward Kdo_2_-lipid A. Finally, LpxE_AA_ also displayed significant activity toward PGP, with a specific activity of 0.75 ± 0.11 μmol/mg/min, ∼40% of its activity toward Kdo_2_-lipid A (Table 1). Taken together, our biochemical assays validate LpxE_AA_ as a trifunctional LPT enzyme that efficiently dephosphorylates chemically diverse Kdo_2_-lipid A (glycolipids), PGP (phosphoglycerol lipid), and C_55_-PP (isoprenyl lipid) *in vitro*.

**TABLE 1:** Specific activities of enzymes in this study

Enzyme	Specific activity (μmol/mg/min)		
	Kdo_2_-lipid A	C_55_-PP	PGP
LpxE_AA_	2.04 ± 0.46	3.58 ± 0.47	0.75 ± 0.11
LpxE_FN_	3.25 ± 0.21	2.99 ± 0.45	0.038 ± 0.009
UppP_FN_	0.010 ± 0.005	22.71 ± 2.62	0.031 ± 0.007

**FIG 3:**
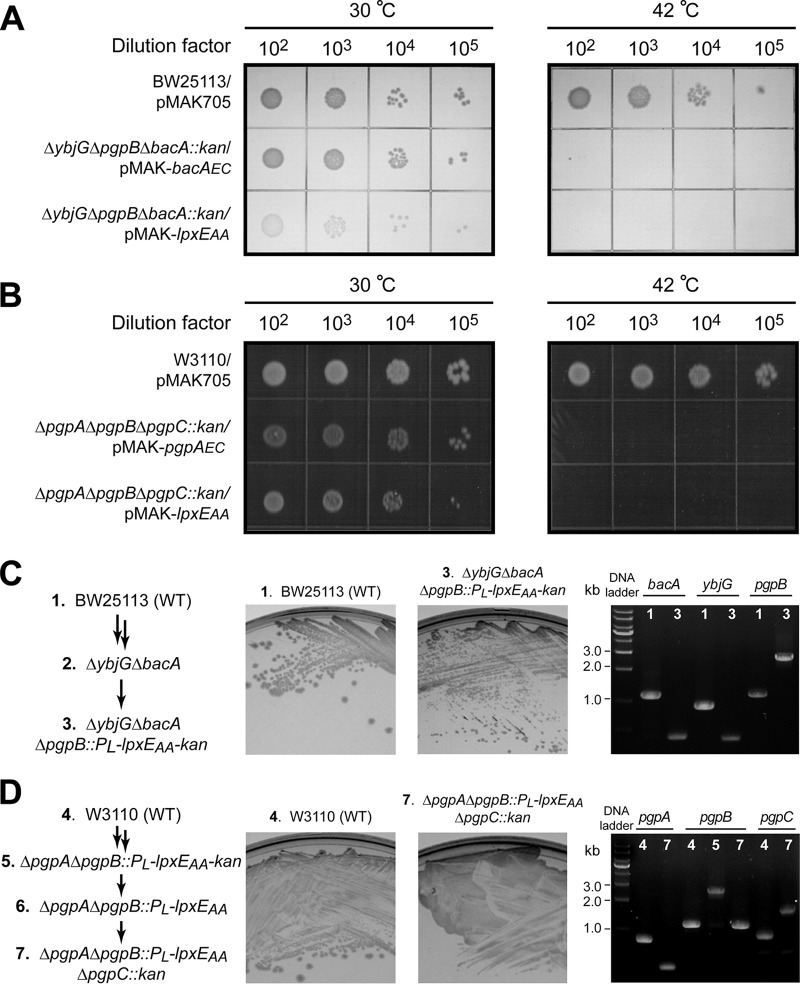
LpxE_AA_ complements E. coli strains deficient in C_55_-PP phosphatase or PGP phosphatase activities. (A) Complementation of the C_55_-PP phosphatase-deficient E. coli strain (BW25113 Δ*ybjG* Δ*pgpB* Δ*bacA*::*kan*) by the temperature-sensitive pMAK705 plasmid harboring *bacA_EC_* (positive control, pMAK-*bacA_EC_*) or *lpxE_AA_* (pMAK-*lpxE_AA_*). WT E. coli cells carrying pMAK705 or C_55_-PP phosphatase-deficient E. coli cells carrying pMAK-*bacA_EC_* or pMAK-*lpxE_AA_* were grown at 30°C or 42°C. From left to right are spots of 10-fold serial dilutions from 10^2^ to 10^5^. (B) Complementation of the PGP phosphatase-deficient E. coli strain (W3110 Δ*pgpA* Δ*pgpB* Δ*pgpC*::*kan*) by the temperature-sensitive pMAK705 plasmid harboring *pgpA_EC_* (positive control, pMAK-*pgpA_EC_*) or *lpxE_AA_* (pMAK-*lpxE_AA_*). WT E. coli cells carrying pMAK705 or PGP phosphatase-deficient E. coli cells carrying pMAK-*pgpA_EC_* or pMAK-*lpxE_AA_* were grown at 30°C or 42°C. From left to right are spots of 10-fold serial dilutions from 10^2^ to 10^5^. (C) Chromosomal complementation of C_55_-PP phosphatase activity-deficient E. coli with *lpxE_AA_*. The left, middle, and right images show the construction of different E. coli C_55_-PP phosphatase gene deletion mutants, the growth of WT E. coli cells and C_55_-PP phosphatase-deficient cells complemented by a chromosomal copy of *lpxE_AA_*, and the PCR verification of *ybjG*, *bacA*, and *pgpB* knockouts of the target mutant strain, respectively. (D) Chromosomal complementation of PGP phosphatase activity-deficient E. coli with *lpxE_AA_*. The left, middle, and right images show the construction of different E. coli PGP phosphatase gene deletion mutants, the growth of WT E. coli cells and PGP phosphatase-deficient cells complemented by a chromosomal copy of *lpxE_AA_*, and PCR verification of *pgpA*, *pgpC*, and *pgpB* knockouts of the target mutant strain, respectively. Since the expected sizes of *pgpB* (1,106 bp) and *pgpB*::*P_L_-lpxE_AA_* (1,093 bp) are similar using primers flanking *pgpB* in the final strain, the knockout of *pgpB* was established by also verifying the PCR result of the mother strain (strain 5: W3110 Δ*pgpA* Δ*pgpB*::*P_L_-lpxE_AA_-frt-kan-frt*).

In order to obtain further evidence of the trifunctional role of LpxE_AA_ in cells, we examined whether LpxE_AA_ could functionally rescue lethal E. coli mutants lacking C_55_-PP phosphatase or PGP phosphatase activities. E. coli contains four C_55_-PP phosphatases, BacA, PgpB, YbjG, and LpxT. A deletion mutant, Δ*ybjG* Δ*bacA* Δ*pgpB*::*kan*, in E. coli is lethal unless rescued by a plasmid expressing BacA, PgpB, or YbjG (26). To examine if LpxE_AA_ could function as a C_55_-PP phosphatase in cells, we set up complementation of the lethal Δ*ybjG* Δ*pgpB* Δ*bacA*::*kan* E. coli mutant carrying *lpxE_AA_* on a low-copy-number, temperature-sensitive pMAK705 vector (pMAK-*lpxE_AA_*). The E. coli *bacA* gene, encoding the C_55_-PP phosphatase, was used as the positive control (pMAK-*bacA_EC_*). We found that overexpression of LpxE_AA_ and BacA_EC_ from pMAK705-derived plasmids complemented the lethal phenotype of the Δ*ybjG* Δ*pgpB* Δ*bacA*::*kan* triple knockout in E. coli on an LB agar plate at 30°C; such a complementation effect was lost when cells were grown at 42°C, consistent with the loss of the temperature-sensitive pMAK705 plasmid encoding LpxE_AA_ or BacA_EC_ and confirming that LpxE_AA_ functionally complements the loss of C_55_-PP phosphatase activity in E. coli (Fig. 3A).

We similarly tested whether LpxE_AA_ functionally complements the loss of PGP phosphatase activity in E. coli. E. coli has three PGP phosphatases, PgpA, PgpB, and PgpC (27). A Δ*pgpA* Δ*pgpB* Δ*pgpC*::*kan* triple-knockout mutant is lethal unless it is rescued by a plasmid harboring an active PGP phosphatase (27). Overexpression of LpxE_AA_ or the positive control PgpA_EC_ from the temperature-sensitive pMAK705 plasmid supported the growth of the Δ*pgpA* Δ*pgpB* Δ*pgpC*::*kan* triple-knockout mutant strains at 30°C but not at 42°C. In contrast, the control strain (W3110/pMAK705) grew well at both temperatures (Fig. 3B). These observations confirm that LpxE_AA_ is a functional PGP phosphatase in E. coli.

While the pMAK705 vector-encoded LpxE_AA_ complemented E. coli triple knockouts lacking C_55_-PP phosphatase or PGP phosphatase activities, pMAK705 has a higher copy number (pSC101 origin, ∼5 copies/cell) than that of the chromosome in E. coli (single copy/cell). In order to mitigate the concern that the observed genetic complementation was caused by multiple copies of the *lpxE_AA_* gene, we replaced the *pgpB* gene in the chromosome of E. coli (BW25113) Δ*ybjG* Δ*bacA* with a gene cassette (*P_L_-lpxE_AA_-FRT-kan-FRT*) containing *lpxE_AA_* and a kanamycin resistance gene under the control of the P_L_ promoter (28). The resulting E. coli strain (E. coli BW25113 Δ*ybjG* Δ*bacA* Δ*pgpB*::*P_L_-lpxE_AA_-FRT-kan-FRT*) grew on an LB agar plate, and the proper knockouts of *bacA*, *ybjG*, and *pgpB* were verified by PCR (Fig. 3C), confirming that the chromosomal copy of *lpxE_AA_* complemented the loss of C_55_-PP phosphatase activity. Using a similar approach, we also replaced the *pgpB* gene of E. coli (W3110) Δ*pgpA* with *P_L_-lpxE_AA_-FRT-kan-FRT*, removed the kanamycin resistance cassette (29), and then knocked out *pgpC*. The resulting strain (E. coli W3110 Δ*pgpA* Δ*pgpB*::*P_L_-lpxE_AA_* Δ*pgpC*::*kan*) also grew on an LB agar plate, and knockouts of *pgpA*, *pgpB*, and *pgpC* were verified by PCR (Fig. 3D), confirming that the chromosomal copy of *lpxE_AA_* similarly complemented the loss of PGP phosphatase activity.

Altogether, the substantial phosphatase activities of LpxE_AA_ toward Kdo_2_-lipid A, C_55_-PP, and PGP *in vitro* and its ability to complement the loss of C_55_-PP and PGP phosphatase activities in E. coli—both via the plasmid-borne gene and via chromosomal knock-in—strongly support the multifunctionality of LpxE_AA_ in Gram-negative bacterial envelope biogenesis.

### LpxE_FN_ is a bifunctional lipid phosphatase *in vitro* and functionally complements an E. coli mutant deficient in the C_55_-PP phosphatase activity.

**FIG 4:**
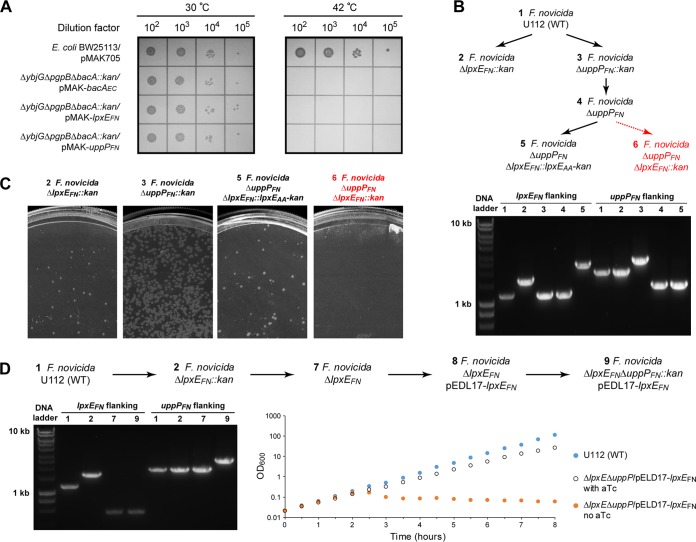
F. novicida harbors two C_55_-PP phosphatases, LpxE_FN_ and UppP_FN_. (A) Complementation of the C_55_-PP phosphatase-deficient E. coli strain (BW25113 Δ*ybjG* Δ*bacA* Δ*pgpB*::*kan*) by the temperature-sensitive pMAK705 plasmid harboring *bacA_EC_* (positive control, pMAK-*bacA_EC_*), *lpxE_FN_* (pMAK-*lpxE_FN_*), or *uppP_FN_* (*ftn_1552*, pMAK-*uppP_FN_*). WT E. coli cells carrying pMAK705 or C_55_-PP phosphatase-deficient E. coli cells carrying pMAK-*bacA_EC_*, pMAK-*lpxE_FN_*, or pMAK-*uppP_FN_* were grown at 30°C or 42°C. From left to right are spots of 10-fold serial dilutions from 10^2^ to 10^5^. (B) Schematic illustration of the construction of different F. novicida gene deletion strains. Viable and lethal strains are in black and red, respectively. The presence of the proper gene deletion was verified by PCR using primers at ∼0.25-kb or 0.6-kb positions flanking *lpxE* or *uppP*, respectively. (C) Viability of F. novicida mutants. While F. novicida mutants containing Δ*lpxE_FN_*::*kan*, Δ*uppP_FN_*::*kan*, or Δ*uppP_FN_* Δ*lpxE_FN_*::*lpxE_AA_* were viable, no colonies could be isolated for F. novicida mutants containing Δ*uppP_FN_* Δ*lpxE_FN_*::*kan*. (D) Construction of the conditional lethal F. novicida strain containing Δ*lpxE_FN_* Δ*uppP_FN_* complemented by aTc-inducible pEDL17-*lpxE_FN_*. The sequence of strain construction is shown at the top. The presence of the desired gene deletion was verified by PCR (left). Prolonged withdrawal of aTc from the growth medium resulted in a slow bactericidal phenotype (right).

Despite the intriguing observation of the multifunctionality of LpxE_AA_, it is challenging to establish the biological consequence in its native host due to the difficulty of culturing and genetic manipulation of A. aeolicus. Therefore, we asked if other LpxE enzymes from genetically trackable bacteria similarly display multifunctional lipid phosphatase activities. In order to answer this question, we chose LpxE_FN_, a distant ortholog of LpxE_AA_, for further characterization. The ability of LpxE_FN_ to dephosphorylate lipid A at the 1-position was previously reported (15), but its activity toward other lipid substrates has not been thoroughly investigated. We first conducted similar complementation experiments using E. coli strains deficient in either the C_55_-PP phosphatase activity or PGP phosphatase activity carrying the temperature-sensitive pMAK-*lpxE_FN_*. We found that LpxE_FN_ complemented the loss of C_55_-PP phosphatase activity of E. coli (Δ*ybjG* Δ*pgpB* Δ*bacA*::*kan*) at 30°C but not at 42°C, indicating that LpxE_FN_ is a functional C_55_-PP phosphatase in E. coli (Fig. 4A). However, we were unable to complement E. coli deficient in the PGP activity (Δ*pgpA* Δ*pgpB* Δ*pgpC*::*kan*) with a plasmid encoding LpxE_FN_ (pMAK-*lpxE_FN_*). Consistently, we found that purified LpxE_FN_ displayed significant phosphatase activity toward both Kdo_2_-lipid A and C_55_-PP and processed these two substrates with similar efficiencies (specific activities of 3.25 ± 0.21 μmol/mg/min for Kdo_2_-lipid A and 2.99 ± 0.45 μmol/mg/min for C_55_-PP), but its activity toward PGP was ∼100-fold lower (specific activity of 0.038 ± 0.009 μmol/mg/min) (Table 1), confirming that LpxE_FN_ is a bifunctional lipid phosphatase.

### F. novicida harbors two C_55_-PP phosphatases: LpxE_FN_ and FTN_1552.

It is important to note that the lipid A 1-phosphatase activity is not essential in bacteria but the C_55_-PP phosphatase activity is. Prior to this study, no enzyme encoding the C_55_-PP phosphatase activity had been identified in F. novicida. As the transposon mutant of *lpxE_FN_* is not lethal in F. novicida (30), we reasoned that there must exist another enzyme encoding the C_55_-PP phosphatase activity in F. novicida. By searching for F. novicida proteins homologous to E. coli enzymes containing C_55_-PP phosphatase activity (i.e., BacA_EC_, YbjG_EC_, PgpB_EC_, and LpxT_EC_) using PSI-BLAST (20), we have identified a PAP2 family protein of unknown function, FTN_1552, as a potential candidate of the C_55_-PP phosphatase (PSI-BLAST of PgpB_EC_: E value of 0.003 and sequence identity of 16.47%). We found that the temperature-sensitive pMAK705 vector harboring *ftn_1552* complemented the E. coli strain deficient in C_55_-PP phosphatase activity (Δ*ybjG* Δ*pgpB* Δ*bacA*::*kan*), confirming *ftn_1552* as the gene encoding the C_55_-PP phosphatase activity (Fig. 4A). FTN_1552 was subsequently renamed UppP_FN_. Purified UppP_FN_ appears to be a specific enzyme for C_55_-PP, with a specific activity of 22.71 ± 2.62 μmol/mg/min, and displays little activity toward Kdo_2_-lipid A and PGP (specific activities of 0.010 ± 0.005 μmol/mg/min and 0.031 ± 0.007 μmol/mg/min, respectively [Table 1]). Importantly, while F. novicida strains containing a chromosomal deletion of either *lpxE_FN_* or *uppP_FN_* were viable, we were unable to generate F. novicida strains containing both deletions (Δ*lpxE_FN_* Δ*uppP_FN_*) in the chromosome (Fig. 4B and C). However, F. novicida cells were viable in the Δ*uppP_FN_* background when *lpxE_FN_* was replaced with *lpxE_AA_* (Fig. 4B and C). Furthermore, when F. novicida was first transformed with a plasmid (pEDL17) bearing *lpxE_FN_* under the control of an anhydrotetracycline (aTc) promoter, we were also able to obtain viable F. novicida colonies containing chromosomal deletions of both *lpxE_FN_* and *uppP_FN_* (U112 Δ*lpxE_FN_* Δ*uppP_FN_*::*kan/*pEDL17*-lpxE_FN_*). The presence of proper chromosomal deletions of the *lpxE_FN_* and *uppP_FN_* genes was verified by PCR (Fig. 4D). As expected, the viability of such a strain depends on the expression of plasmid-encoded LpxE_FN_: prolonged withdrawal of aTc suppressed the bacterial growth and slowly resulted in cell lysis in culture (Fig. 4D), reinforcing the notion that UppP_FN_ and LpxE_FN_ share redundant C_55_-PP phosphatase activities in *Francisella*.

### LpxE_FN_ functionally connects multiple layers of envelope biogenesis in F. novicida.

**FIG 5:**
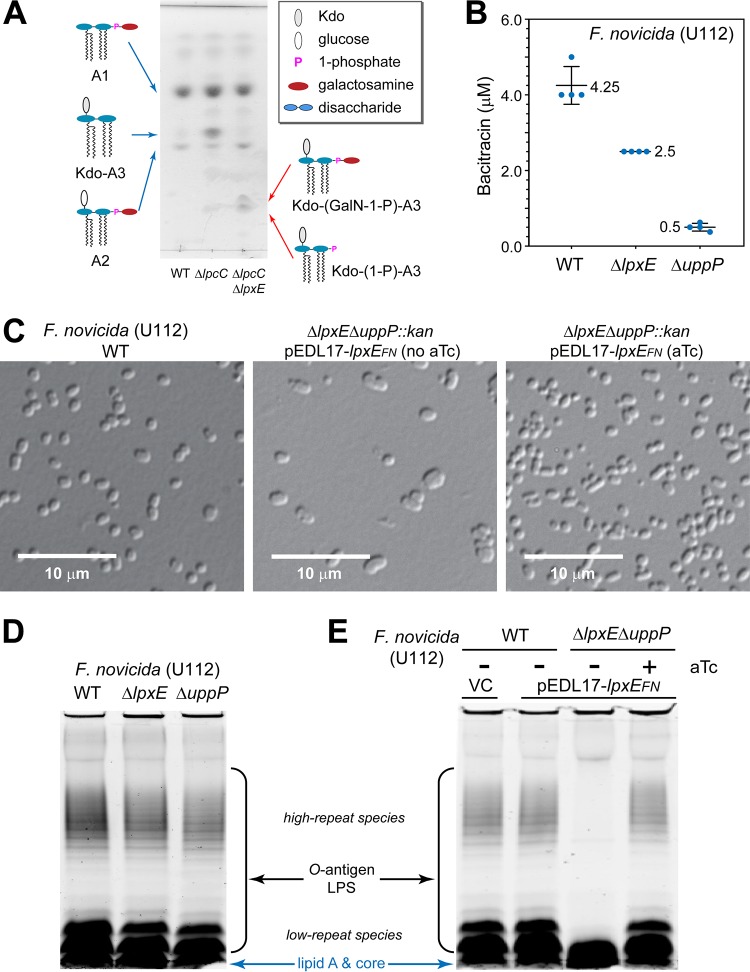
LpxE_FN_ functionally contributes to multiple layers of the F. novicida bacterial envelope biogenesis. (A) Deletion of *lpxE_FN_* results in accumulation of 1-phosphorylated Kdo-lipid A3 species. The profiles of total lipid extracts from F. novicida U112 WT, Δ*lpcC*, and Δ*lpcC* Δ*lpxE* strains were analyzed by TLC. Lipid A species are labeled. Abbreviations: GalN, galactosamine; A1, lipid A1; A2, lipid A2; Kdo-A3, Kdo-lipid A3; Kdo-(GalN-1-P)-A3, Kdo-(galactosamine-1-phospho)-lipid A3; Kdo-(1-P)-A3, Kdo-(1-phospho)-lipid A3. (B) Deletion of *lpxE_FN_* sensitizes F. novicida to bacitracin as reflected by reduced MIC. Error bars represent standard deviations from quadruplet measurements. (C) Suppression of the plasmid-encoded LpxE_FN_ expression in the Δ*lpxE_FN_* Δ*uppP_FN_*::*kan* mutant of F. novicida causes cell deformation. Images of wild-type cells and Δ*lpxE_FN_* Δ*uppP_FN_*::*kan* F. novicida mutant cells without and with LpxE_FN_ expression are shown in the left, middle, and right images, respectively. (D) Lack of LPS phenotypes in F. novicida cells containing the single deletion of *lpxE_FN_* or *uppP_FN_*. (E) Suppression of the plasmid-encoded LpxE_FN_ expression in the Δ*lpxE_FN_* Δ*uppP_FN_*::*kan* mutant of F. novicida causes the loss of O-antigen repeats in LPS. LPS profiles from wild-type F. novicida cells carrying the pDEL17 vector (VC) or the C_55_-PP phosphatase-deficient (Δ*lpxE_FN_* Δ*uppP_FN_*::*kan*) cells carrying the pDEL17-*lpxE_FN_* vector without or with aTc were analyzed on SDS-PAGE gels using the Pro-Q Emerald LPS staining kit. O-antigen-containing LPS, including both high- and low-repeat species, and free lipid A/core species are labeled.

After establishing that LpxE_FN_ shares C_55_-PP phosphatase activity with UppP_FN_, we further examined the biological implication of the multifunctional enzymatic activity of LpxE_FN_ in its native host, F. novicida. We first verified the role of LpxE_FN_ as a lipid A 1-phosphatase. Wild-type (WT) F. novicida cells contain both LPS (i.e., core oligosaccharide and O-antigen-modified Kdo-lipid A3 without 1- and 4′-phosphates) and free lipid A species A1 and A2, which do not contain core oligosaccharides/Kdo or O-antigen (lipid A2 differs from lipid A1 in that it has an additional α-linked glucose moiety attached to its 6′-position; also see the schematic lipid A structures of WT F. novicida in Fig. 5A) (31). Both lipid A1 and lipid A2 are further modified by FlmK, which transfers galactosamine from C_55_-P-galactosamine to the 1-phosphate of lipid A (32, 33). As the core oligosaccharide and O-antigen-modified lipid A are inefficiently extracted by the Bligh-Dyer method for mass spectrometry analysis, we examined the effect of Δ*lpxE_FN_* in the F. novicida strain deficient in the glycosyltransferase activity (Δ*lpcC*), which produces Kdo-lipid A3 (instead of LPS), in addition to lipids A1 and A2 found in the wild-type cells (Fig. 5A). Accumulations of Kdo-(1-phospho)-lipid A3 and Kdo-(galactosamine-1-phospho)-lipid A3, as well as the disappearance of Kdo-lipid A3, were observed in F. novicida when *lpxE_FN_* was deleted (Δ*lpxE_FN_*), confirming the lipid A 1-phosphatase activity of LpxE_FN_ in cells (Fig. 5A and Fig. S5).

10.1128/mBio.00886-19.5 FIG S5 Mass spectrometry analysis of purified lipid A species of F. novicida mutants from preparative TLC. Accumulated new lipid A species from F. novicida mutants (Δ*lpcC*::*tet* and Δ*lpxE*::*kan* Δ*lpcC*::*tet*) isolated from TLC are identified using mass spectrometry as Kdo-lipid A3 (A), Kdo-(galactosamine-1-phospho)-lipid A3 (B), and Kdo-(1-phospho)-lipid A3 (C). Download FIG S5, TIF file, 2.8 MB. Copyright © 2019 Zhao et al. 2019 Zhao et al. This content is distributed under the terms of the Creative Commons Attribution 4.0 International license.

As the C_55_-PP phosphatase activity of LpxE_FN_ (2.99 ± 0.45 μmol/mg/min) is only ∼7-fold smaller than that of UppP_FN_ (22.71 ± 2.62 μmol/mg/min), we asked whether LpxE_FN_ could functionally contribute to the bacterial envelope biogenesis beyond lipid A modification at the 1-phosphate position. We first compared the sensitivities of the wild-type F. novicida strain (U112) and mutant strains containing either the *lpxE* or *uppP* deletion to bacitracin, an antibiotic sequestering C_55_-PP. We found that while the loss of *uppP* in F. novicida generated an 8.5-fold drop of MIC in comparison with that of the WT strain (0.5 μM versus 4.25 μM), as expected, the loss of *lpxE* also resulted in ∼1.7-fold drop of the MIC of bacitracin (2.5 μM for F. novicida Δ*lpxE*) (Fig. 5B), implicating a functional role of LpxE_FN_ in the recycling of C_55_-PP.

In order to isolate the biological effect of LpxE_FN_, we utilized the F. novicida strain containing chromosomal deletions of both *uppP_FN_* and *lpxE_FN_*, which is complemented by a plasmid carrying *lpxE_FN_* under the control of an aTc promoter. We found that the loss of plasmid-mediated expression of LpxE_FN_ due to withdrawal of aTc in the growth medium resulted in cell enlargement, reflecting defective peptidoglycan biosynthesis (Fig. 5C). Strikingly, while no change of O-antigen repeats was observed in F. novicida cells containing the chromosomal deletion of either *lpxE* or *uppP* in comparison with WT cells (Fig. 5D), transient suppression of LpxE led to a dramatic reduction of the LPS O-antigen repeats, including both high- and low-repeat species (Fig. 5E) (34), suggesting a contribution of LpxE to the O-antigen biogenesis. These observations are consistent with the notion that the biosynthesis and transport of peptidoglycan and O-antigen depend on C_55_-P, the product of LpxE_FN_ (and UppP_FN_) activity, and reveal a previously unappreciated function of LpxE in the biogenesis and remodeling of multiple components across the bacterial envelope: peptidoglycan, free lipid A, and the O-antigen repeat of LPS.

## DISCUSSION

LpxE enzymes are important virulence factors that promote bacterial survival, fitness, and pathogenicity. In H. pylori and Rhizobium etli CE3, the chromosomal knockout of *lpxE* resulted in increased susceptibility to positively charged antimicrobial peptides such as polymyxin B and colistin (9, 35), presumably due to the retention of 1-phosphate of lipid A. Previous studies showed that *Rhizobium* LpxE displays over a 1,000-fold preference of Kdo_2_-lipid A/lipid IV_A_ over PGP; therefore, LpxE has been regarded as a highly specific monofunctional enzyme whose sole activity is to remove the 1-phosphate from lipid A. In this study, based on the striking structural similarity between LpxE_AA_ and YodM_BS_, a PGP phosphatase with a weak *in vitro* activity on C_55_-PP phosphatase, we discovered that LpxE is a multifunctional lipid phosphatase. The LpxE enzyme from A. aeolicus displays significant activities toward Kdo_2_-lipid A/lipid IV_A_, C_55_-PP and PGP and functionally complements E. coli strains deficient in C_55_-PP or PGP phosphatase activities. Likewise, the LpxE enzyme from F. novicida is a dual-function enzyme that processes Kdo_2_-lipid A and C_55_-PP with similar efficiencies. Strikingly, deletion of *lpxE_FN_* in its native host F. novicida resulted in accumulation of phosphorylated lipid A species and increased sensitivity to bacitracin; in the C_55_-PP phosphatase-deficient (Δ*uppP* and Δ*lpxE* double-knockout mutant) F. novicida, suppression of LpxE_FN_ expression from the plasmid resulted in cell deformation due to defective peptidoglycan biosynthesis and the loss of O-antigen repeats in LPS associated with reduced O-antigen transport, both of which are critically dependent on the recycling of C_55_-PP to C_55_-P. Taken together, these results show that LpxE enzymes from A. aeolicus and F. novicida functionally connect multiple layers of bacterial envelope biogenesis and remodeling. Such multiple functional roles are not unique to LpxE enzymes from A. aeolicus and F. novicida: we found that LpxE enzymes from H. pylori and R. leguminosarum also complemented E. coli deficient in C_55_-PP phosphatase activities (Fig. S6), suggesting that these LpxE enzymes can similarly process Kdo_2_-lipid A and C_55_-PP to synchronize lipid A modification with peptidoglycan biosynthesis and O-antigen modification of LPS.

10.1128/mBio.00886-19.6 FIG S6 Complementation of the C_55_-PP phosphatase-deficient E. coli strain by LpxE enzymes from R. leguminosarum (LpxE_RL_) and H. pylori (LpxE_HP_). Wild-type E. coli cells carrying an empty vector (BW25113/pMAK705) or C_55_-PP phosphatase-deficient E. coli cells (BW25113 Δ*ybjG* Δ*pgpB* Δ*bacA*::*kan*) carrying pMAK-*lpxE_RL_* or *lpxE_HP_* were grown at 30°C or 42°C, respectively. From left to right are spots of 10-fold serial dilutions from 10^2^ to 10^5^. Download FIG S6, TIF file, 2.2 MB. Copyright © 2019 Zhao et al. 2019 Zhao et al. This content is distributed under the terms of the Creative Commons Attribution 4.0 International license.

**FIG 6:**
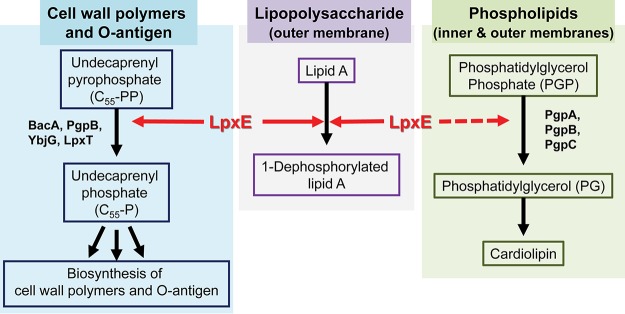
Multiple roles of LpxE in the biogenesis of the Gram-negative bacterial envelope. Shown is a schematic illustration of the multifunctional roles of LpxE in the biogenesis of the cell wall polymer (peptidoglycan), outer membrane (LPS), and inner membrane (PGP). Shared phosphatase activities of LpxEs from diverse bacteria, including *Aquifex* (*Aquificales*), *Francisella* (*Gammaproteobacteria*), *Rhizobium* (*Alphaproteobacteria*), and *Helicobacter* (*Epsilonproteobacteria*), toward lipid A and C_55_-PP are indicated by solid arrows, whereas the unique PGP phosphatase activity of LpxE_AA_ is indicated by a dashed red line.

It is appropriate to ask why LpxE has evolved into a multifunctional enzyme. There are several potential explanations. First, it is conceivable that the peptidoglycan biosynthesis is such an essential process that multiple enzymes, including LpxE, are employed as the backup enzymes for the C_55_-PP phosphatase-mediated recycling reaction for peptidoglycan charging and biosynthesis. Second, it is possible that LpxE from *Aquifex* species represents an ancestral lipid phosphatase, which, although primitive, is sufficient to conduct all lipid phosphatase activities to support the bacterial envelope biogenesis and remodeling, while other LPT family of lipid phosphatases, such as the PGP phosphatase, evolved later as specialized, highly efficient enzymes. Third, it is also likely that LpxE evolved as a multifunctional enzyme to coordinate lipid A modification and the biogenesis of other layers of bacterial envelope. As 1,4′-bisphosphorylated lipid A chelates metal ions to form a fortified layer for bacterial protection, removal of the 1-phosphate could weaken the lipid A layer and increase membrane permeability. It is conceivable that the weakened lipid A layer is compensated by the elevated peptidoglycan biosynthesis and enhanced O-antigen decoration of LPS. Thus, bestowing LpxE with the multifunctionality toward Kdo_2_-lipid A and C_55_-PP (and, in the case of A. aeolicus, PGP) enables LpxE to orchestrate lipid A modification with bacterial envelope remodeling at multiple layers (Fig. 6) in order to promote the optimal bacterial growth and enhance bacterial survival in nature and the human host.

The Gram-negative bacterial envelope contains three layers. How Gram-negative bacteria coordinate the biogenesis and remodeling of different layers of the bacterial envelope has remained an area of active investigation. Our study has revealed the first biological evidence of a multifunctional enzyme, LpxE in F. novicida, that natively couples lipid A 1-dephosphorylation with C_55_-PP recycling to enhance peptidoglycan biogenesis and O-antigen decoration of LPS, promote cell viability against antimicrobial peptides, evade host immune surveillance, and ultimately support bacterial pathogenesis. We suggest that such a multifunctional role represents a common but previously unappreciated mechanism for Gram-negative bacteria to coordinate bacterial envelop biogenesis across different layers.

## MATERIALS AND METHODS

Data collection and refinement statistics of LpxE_AA_ are listed in Table S1. All strains and plasmids used in this work are listed in Tables S2 and S3, respectively.

10.1128/mBio.00886-19.8 TABLE S2 Plasmids used in this work. Download Table S2, DOCX file, 0.02 MB. Copyright © 2019 Zhao et al. 2019 Zhao et al. This content is distributed under the terms of the Creative Commons Attribution 4.0 International license.

10.1128/mBio.00886-19.9 TABLE S3 Strains used in this work. Download Table S3, DOCX file, 0.02 MB. Copyright © 2019 Zhao et al. 2019 Zhao et al. This content is distributed under the terms of the Creative Commons Attribution 4.0 International license.

Plasmid and strain constructions and growth conditions are described in the supplemental material in detail.

Extraction of lipid A species, TLC and mass spectrometry analyses of lipid A species, and assay conditions are described in the Supplementary Methods section of the supplemental material.

Characterizations of F. novicida U112 mutants are described in the Supplementary Methods section of the supplemental material.

10.1128/mBio.00886-19.10 TEXT S1 Supplemental methods. Download Text S1, DOCX file, 0.1 MB. Copyright © 2019 Zhao et al. 2019 Zhao et al. This content is distributed under the terms of the Creative Commons Attribution 4.0 International license.
