# Supplementary figures and images for: The Lipid A 1-Phosphatase, LpxE, Functionally Connects Multiple Layers of Bacterial Envelope Biogenesis
Source: mBio. 2019 Jun 18;10(3):e00886-19. doi: 10.1128/mBio.00886-19 (PMC6581854; doi:10.1128/mBio.00886-19)

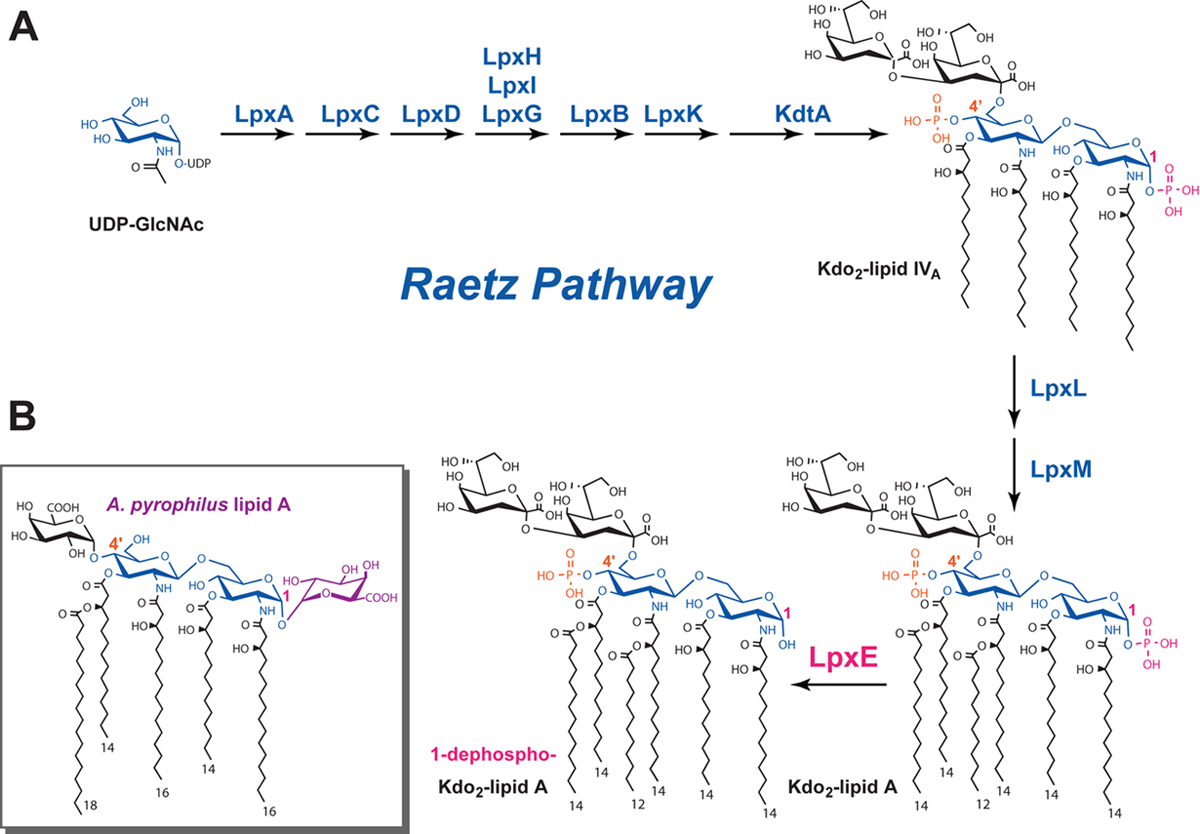

Supplement: FIG S1 [file mBio.00886-19-sf001.tif]

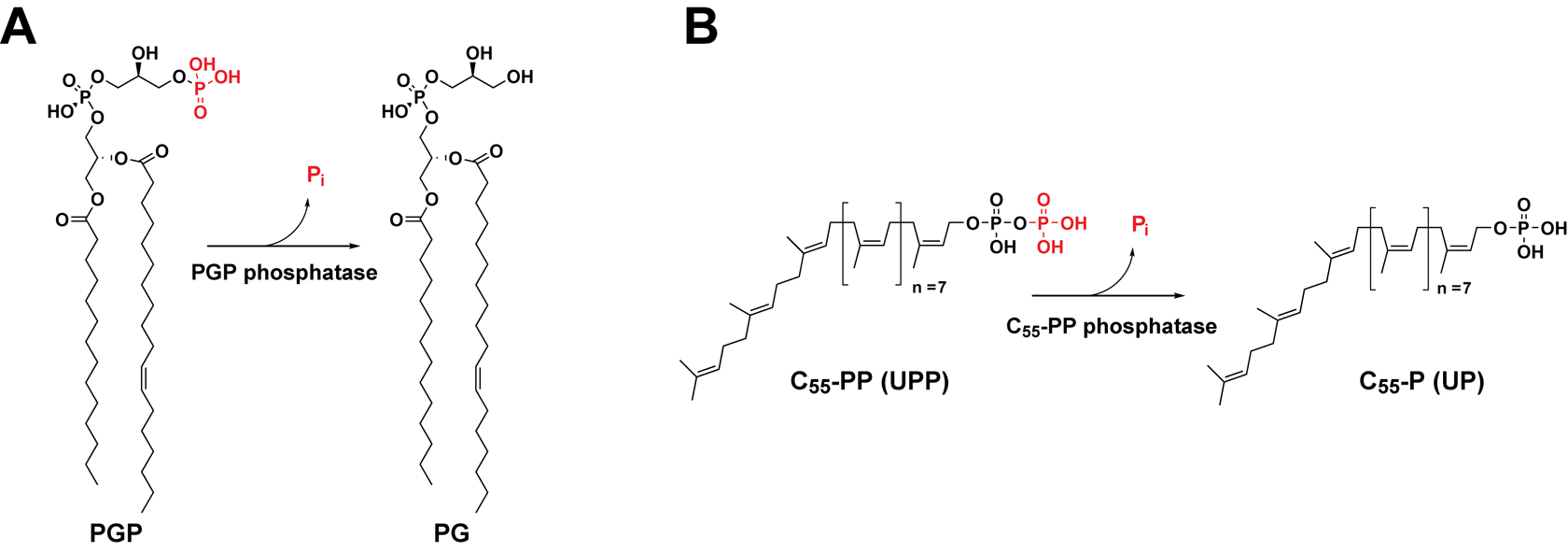

Supplement: FIG S2 [file mBio.00886-19-sf002.tif]

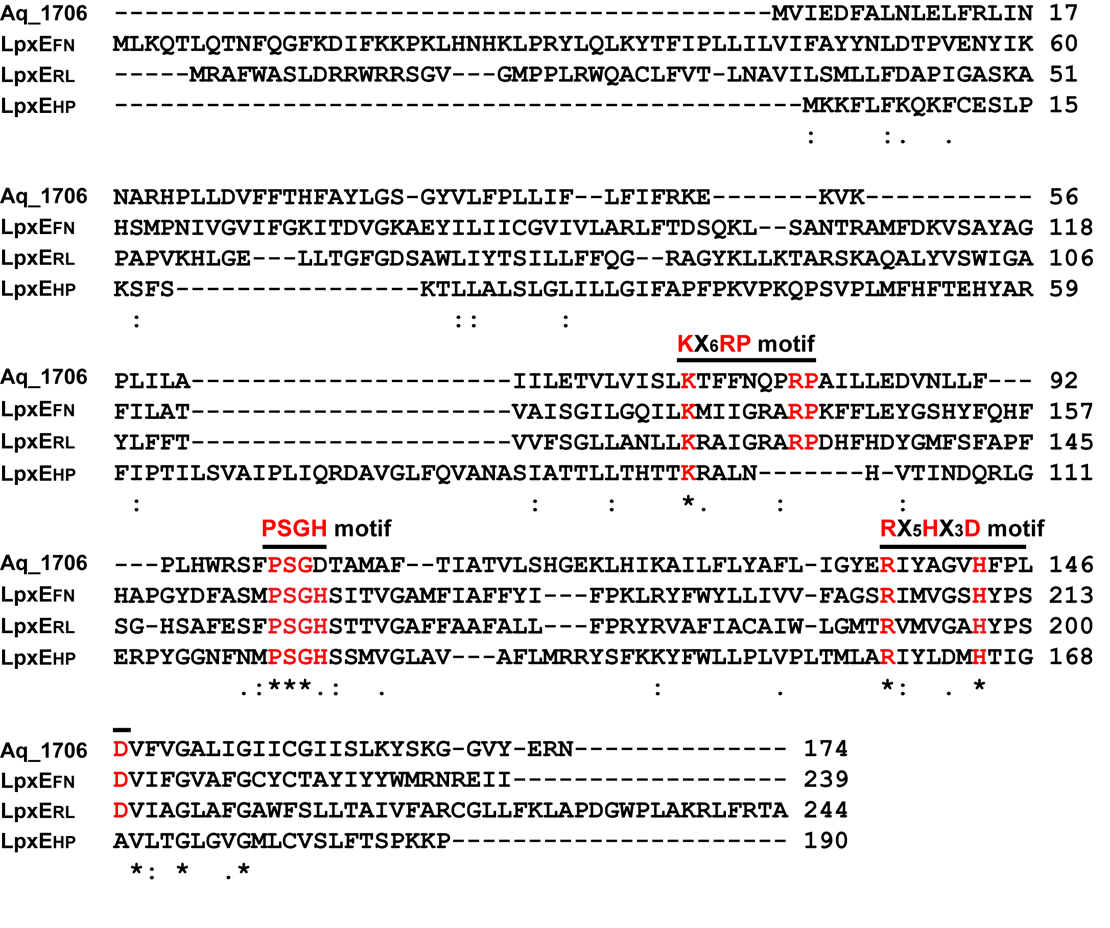

Supplement: FIG S3 [file mBio.00886-19-sf003.tif]

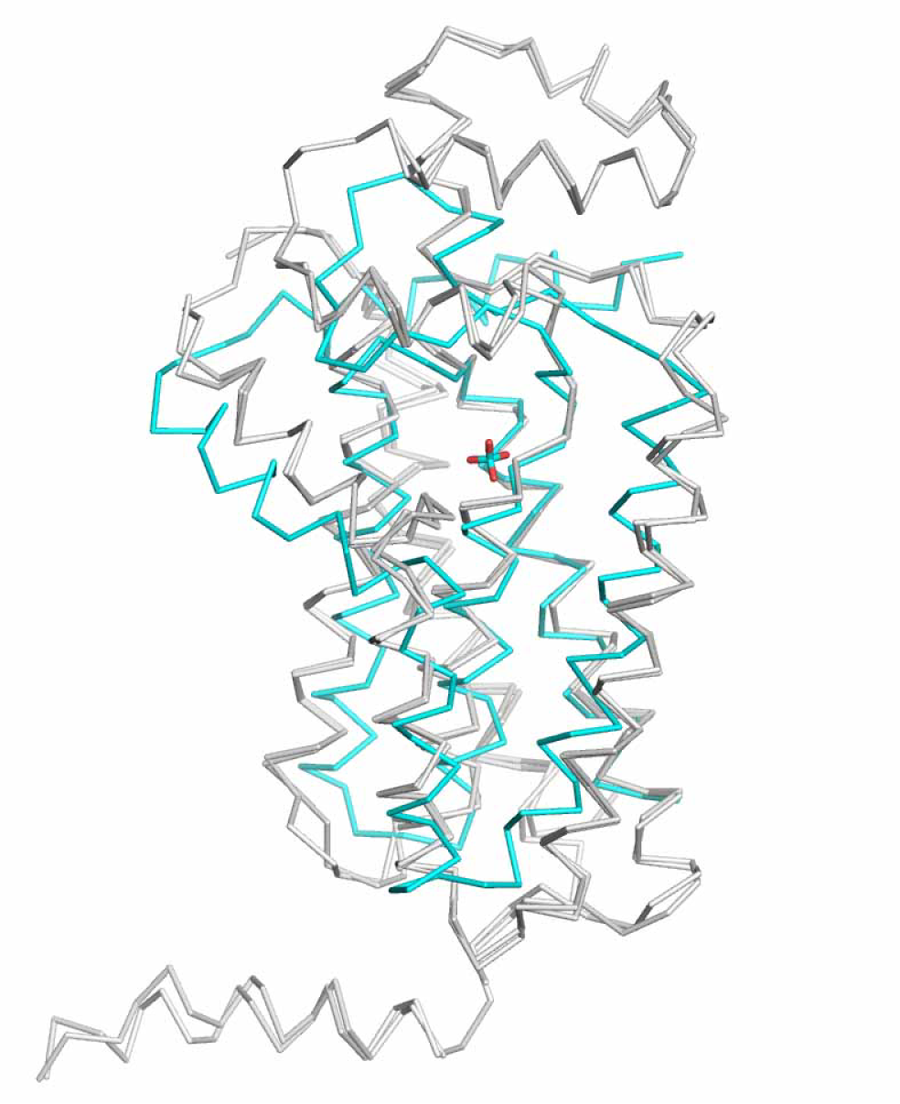

Supplement: FIG S4 [file mBio.00886-19-sf004.tif]

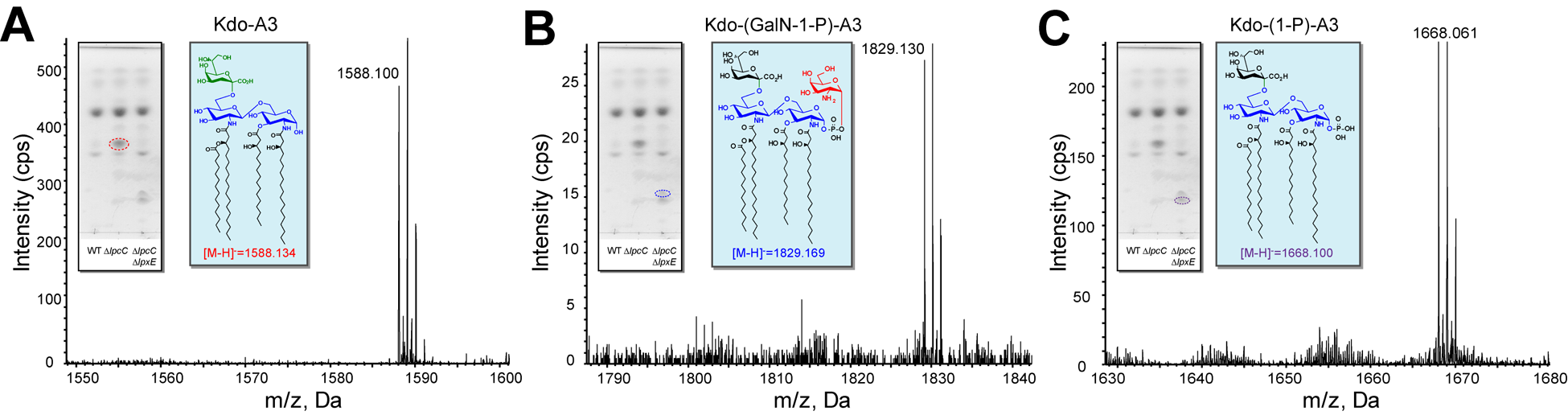

Supplement: FIG S5 [file mBio.00886-19-sf005.tif]

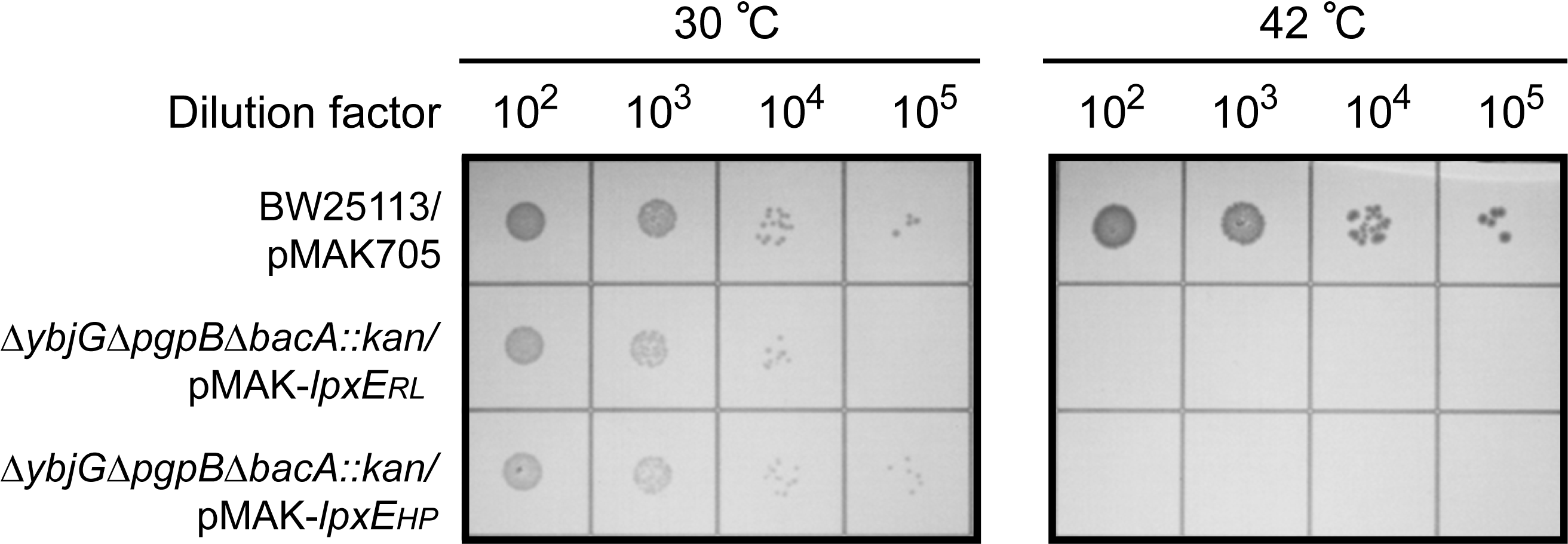

Supplement: FIG S6 [file mBio.00886-19-sf006.tif]
