# Supplementary material for: The Lipid A 1-Phosphatase, LpxE, Functionally Connects Multiple Layers of Bacterial Envelope Biogenesis
Source: mBio. 2019 Jun 18;10(3):e00886-19. doi: 10.1128/mBio.00886-19 (PMC6581854; doi:10.1128/mBio.00886-19)
Supplement: TABLE S1 [file mBio.00886-19-st001.docx]

**Supplementary Table S1:** **Data collection and refinement statistics of LpxE_AA_**

|  | **MR-Se-SAD** | **Native** |
| --- | --- | --- |
| **Data collection** |  |  |
| Wavelength (Å) | 0.9791 | 0.9792 |
| Space group | C 2 2 21 | C 2 2 21 |
| Cell dimensions |  |  |
| *a*, *b*, *c* (Å) | 78.95, 79.68, 82.05 | 80.03, 80.20, 80.44 |
| α, β, γ (°) | 90, 90, 90 | 90, 90, 90 |
| Resolution (Å) | 39.84 – 3.14 (3.22 – 3.14) | 46.32 – 2.37 (2.46 – 2.37) |
| *R*_meas_ | 0.116 (0.823) | 0.095 (1.142) |
| Mean *I*/σ*I* | 20.87 (3.05) | 14.03 (1.84) |
| Completeness (%) | 100 (100) | 99.19 (93.91) |
| Redundancy | 13.75 (14.67) | 6.3 (5.2) |
| Total reflections | 65444 (4929) | 67383 (5206) |
| Unique reflections | 4757 (336) | 10720 (1002) |
|  |  |  |
| **MR-SAD Phasing** |  |  |
| Figure of Merit (FOM) | 0.396 |  |
|  |  |  |
| **Refinement** |  |  |
| *R*_work_ / *R*_free_ |  | 0.208/0.231 |
| No. atoms |  | 1442 |
| Protein |  | 1304 |
| Ligand/ion |  | 77 |
| Water |  | 61 |
| Average *B*-factors |  | 46.21 |
| Protein |  | 45.08 |
| Ligand/ion |  | 56.09 |
| Water |  | 58.00 |
| R.m.s. deviations |  |  |
| Bond lengths (Å) |  | 0.004 |
| Bond angles (°) |  | 0.92 |
| Ramachandran |  |  |
| Favored (%) |  | 96.93 |
| Allowed (%) |  | 3.07 |
| Outliers (%) |  | 0.0 |

*Values in parentheses are for highest-resolution shell.
