# Supplementary material for: The Lipid A 1-Phosphatase, LpxE, Functionally Connects Multiple Layers of Bacterial Envelope Biogenesis
Source: mBio. 2019 Jun 18;10(3):e00886-19. doi: 10.1128/mBio.00886-19 (PMC6581854; doi:10.1128/mBio.00886-19)
Supplement: TABLE S2 [file mBio.00886-19-st002.docx]

**Supplementary Table S2: Plasmids used in this work**

| **plasmid** | **Description** | **Cloning sites** | | **Source** | |  |
| --- | --- | --- | --- | --- | --- | --- |
| pWSK29 | Low copy vector, *lac* promoter, Amp^R^ | |  | | (25) | |
| pMAK705 | Low copy vector, *lac* promoter, ori101, rep 101 Ts, Cam^R^ | |  | | (26) | |
| pET21b | Expression vector, T7 promoter, Amp^R^ | |  | | Novagen | |
| pET26b | Expression vector, T7 promoter,  Kan^R^ | |  | | Novagen | |
| pTRCHis | Expression vector, pTrc promoter,  Amp^R^ | |  | | ThermoFisher | |
| pEDL17 | TetR based inducible vector, Hyg^R^, *FTRp*-mOrange2, *rpsLp-tetR* | |  | | (21) | |
| pLPXE-4 | pET28(a) harboring *R. leguminosarum lpxE* | |  | | (27) | |
| pET21-*lpxE_AA_* | pET21(b) harboring *A. aelicus lpxE (aq_1706)* | | Nde/XhoI | | This work | |
| pET21-*pgsA_EC_* | pET21(b) harboring *E. coli pgsA* | | NdeI/XhoI | | This work | |
| pET16-*uppS_EC_* | pET16(b) harboring *E. coli uppS* | | NdeI/BamHI | | This work | |
| pWSK29-*lpxE_AA_* | pWSK29 harboring *A. aelicus lpxE* | | XbaI/XhoI | | This work | |
| pMAK-*bacA_EC_* | pMAK705 harboring *E. coli bacA* | | HindIII/BamHI | | This work | |
| pMAK-*lpxE_AA_* | pMAK705 harboring *A. aelicus lpxE* | | HindIII/BamHI | | This work | |
| pMAK-*lpxE_FN_* | pMAK705 harboring *F. novicida lpxE* | | HindIII/BamHI | | This work | |
| pMAK-*lpxE_RL_* | pMAK705 harboring *R. leguminosarum lpxE* | | HindIII/BamHI | | This work | |
| pMAK-*lpxE_HP_* | pMAK705 harboring *H. pylori lpxE* | | HindIII/BamHI | | This work | |
| pMAK- *uppP_FN_* | pMAK705 harboring *F. novicida ftn_1552* | | HindIII/BamHI | | This work | |
| pBAD30-*lpxE_AA_* | pBAD30 harboring *A. aelicus lpxE* | | XbaI/HindIII | | This work | |
| pBAD30-*lpxE_AA_-FRT-kan-FRT* | pBAD30 harboring *lpxE_AA_-FRT-kan-FRT* | | HindIII | | This work | |
| pPLpro-*lpxE_AA_-FRT-kan-FRT* | *araC* and P_BAD_ of pBAD30-*lpxE_AA_-FRT-kan-FRT* were replaced with P_L_ promoter | |  | | This work | |
| pEDL17-*lpxE_FN_* | pEDL17 harboring *F. novicida lpxE* (replacing *mOrange2*) | | N/A | | This work | |
| pET26b-pelB-His-MBP-*lpxE_AA_* | pET26b expression construct of pelB-His_10_-MBP-LpxE_AA_ | | NdeI/XhoI | | This work | |
| pTRC-HisMBP-*lpxE_FN_*-strep | pTRCHis expression construct of His_10_-MBP-LpxE_FN_-strep | | NcoI/XhoI | | This work | |
| pTRC-HisMBP-*uppP_FN_* | pTRCHis expression construct of His_10_-MBP-UppP_FN_ | | NcoI/XhoI | | This work | |
