# Supplementary material for: The Lipid A 1-Phosphatase, LpxE, Functionally Connects Multiple Layers of Bacterial Envelope Biogenesis
Source: mBio. 2019 Jun 18;10(3):e00886-19. doi: 10.1128/mBio.00886-19 (PMC6581854; doi:10.1128/mBio.00886-19)
Supplement: TABLE S3 [file mBio.00886-19-st003.docx]

**Supplementary Table S3: Strains used in this work**

| **Strain** | **Description** | **Source** |
| --- | --- | --- |
| W3110 | Wild type F^-^ λ^-^ *rph-1* *IN*(*rrnD*, *rrnE*) | *E. coli* Genetic Stock Center, Yale |
| BW25113 | Wild type *rrnB3* Δ*lacZ4787 hsdR514* Δ(*araBAD*)567 Δ(*rhaBAD*)568 *rph-1* | *E. coli* Genetic Stock Center, Yale |
| C41(DE3) | F^-^ *ompT hsdS_B_ (r_B_^-^m_B_^-^) gal dcm* (DE3) | (25) |
| WBB06 | W3110 *mtl* (Δ*waaC-waaF*)::*tet6* heptose deficient LPS | (1) |
| DH5α | F^-^ *endA1 glnV44 thi-1 recA1 relA1 gyrA96 deoR nupG Φ80dlacZΔM15 Δ(lacZYA-argF)U169*, *hsdR17(r_K_^-^ m_K_^+^), λ–* | (26) |
| U112 | Wild-type F. novicida | Dr. F. Nano, Univ. of Victoria, Canada |
| JW1270 | BW25113 Δ*pgpB::kan* | (14) |
| JW3029 | BW25113 Δ*bacA::kan* | (14) |
| JW5112 | BW25113 Δ*ybjG::kan* | (14) |
| JW5408 | BW25113 Δ*pgpC::kan* | (14) |
| YL10 | W3110 Δ*pgpB* Δ*pgpA* | (3) |
| YL24/ pMAK-*pgpA_EC_* | W3110 Δ*pgpB*Δ*pgpA*Δ*pgpC::kan* pMAK- *pgpA_EC_,* previously named as YL24/pMAK-A | (3) |
| KHSC0007 | BW25113 Δ*ybjG* | This work |
| KHSC0008 | BW25113 Δ*ybjG* Δ*pgpB::kan* | This work |
| KHSC0009 | BW25113 Δ*ybjG* Δ*pgpB* | This work |
| BW25113/pMAK705 | BW25113 pMAK705 | This work |
| KHSC0009/  pMAK-*bacA_EC_* | KHSC0009 carrying pMAK-*bacA_EC_* | This work |
| KHSC0009/  pMAK-*lpxE_AA_* | KHSC0009 carrying pMAK-*lpxE_AA_* | This work |
| KHSC0009/  pMAK-*lpxE_FN_* | KHSC0009 carrying pMAK-*lpxE_FN_* | This work |
| KHSC0009/  pMAK-*lpxE_RL_* | KHSC0009 carrying pMAK-*lpxE_RL_* | This work |
| KHSC0009/  pMAK-*lpxE_HP_* | KHSC0009 carrying pMAK-*lpxE_HP_* | This work |
| KHSC0009/  pMAK-*uppP_FN_* | KHSC0009 carrying pMAK- *uppP_FN_* | This work |
| KHSC0010/  pMAK-*bacA_EC_* | BW25113 Δ*ybjG*Δ*pgpB*Δ*bacA::kan* pMAK-*bacA_EC_*; derived from KHSC0009/pMAK-*bacA_EC_* | This work |
| KHSC0010/  pMAK-*lpxE_AA_* | BW25113 Δ*ybjG*Δ*pgpB*Δ*bacA::kan* pMAK-*lpxE_AA_*; derived from KHSC0009/pMAK-*lpxE_AA_* | This work |
| KHSC0010/  pMAK-*lpxE_FN_* | BW25113 Δ*ybjG*Δ*pgpB*Δ*bacA::kan* pMAK-*lpxE_FN_*; derived from KHSC0009/pMAK-*lpxE_FN_* | This work |
| KHSC0010/  pMAK-lpxE_RL_ | BW25113 Δ*ybjG*Δ*pgp*BΔbacA::kan pMAK-lpxERL; derived from KHSC0009/pMAK-lpxERL | This work |

Continued

**Supplementary Table S3 (continued): Strains used in this work**

| **Strain** | **Description** | **Source** |
| --- | --- | --- |
| KHSC0010/  pMAK-*lpxE_HP_* | BW25113 Δ*ybjG*Δ*pgpB*Δ*bacA::kan* pMAK-*lpxE_HP_*; derived from KHSC0009/pMAK-*lpxE_HP_* | This work |
| KHSC0010/  pMAK-*ftn_1552* | BW25113 Δ*ybjG*Δ*pgpB*Δ*bacA::kan* pMAK-*ftn_1552*; derived from KHSC0009/pMAK-*uppP_FN_* | This work |
| W3110/  pMAK705 | W3110 carrying pMAK705 | This work |
| YL10/  pMAK-*lpxE_AA_* | YL10 carrying pMAK-*lpxE_AA_* | This work |
| YL10/  pMAK-*lpxE_FN_* | YL10 carrying pMAK-*lpxE_FN_* | This work |
| YL24/  pMAK-*lpxE_AA_* | W3110 Δ*pgpB*Δ*pgpA*Δ*pgpC::kan* pMAK-*lpxE_AA_*; derived from pMAK-*lpxE_AA_*/YL10 | This work |
| KAJS0003 | DY330 Δ*pgpB::P_L_-lpxE-FRT-kan-FRT* | This work |
| KAJS0004 | BW25113 Δ*ybjG*Δ*bacA* | This work |
| KAJS0005 | BW25113 Δ*ybjG*Δ*bacA*Δ*pgpB::P_L_-lpxE-FRT-kan-FRT* | This work |
| KAJS0006 | W3110 Δ*pgpA*Δ*pgpB::P_L_-lpxE-FRT-kan-FRT* | This work |
| KAJS0007 | W3110 Δ*pgpA* | This work |
| KAJS0008 | W3110 Δ*pgpA*Δ*pgpB::P_L_-lpxE* | This work |
| KAJS0009 | W3110 Δ*pgpA*Δ*pgpB::P_L_-lpxE*Δ*pgpC::kan* | This work |
| XWK1 | U112 Δ*lpxE::kan* | Kindly provided by Dr. Xiaoyuan Wang |
| JZ1801 | U112 Δ*lpcC::tet* | This work |
| JZ1802 | U112 Δ*lpxE::kan*Δ*lpcC::tet* | This work |
| JZ1803 | U112 Δ*lpxE::FRT-kan-FRT* | This work |
| JZ1804 | U112 Δ*lpxE* | This work |
| JZ1805 | U112 Δ*uppP* (*ftn_1552*)*::FRT-kan-FRT* | This work |
| JZ1806 | U112 Δ*uppP* (*ftn_1552*) | This work |
| JZ1807 | U112 Δ*uppP* (*ftn_1552*) Δ*lpxE::pelB-lpxE_AA_-FRT-kan-FRT* | This work |
| JZ1808 | U112 Δ*lpxE*Δ*uppP* (*ftn_1552*)*::FRT-kan-FRT*/pEDL17-*lpxE_FN_* | This work |
