# Supplementary material for: The Lipid A 1-Phosphatase, LpxE, Functionally Connects Multiple Layers of Bacterial Envelope Biogenesis
Source: mBio. 2019 Jun 18;10(3):e00886-19. doi: 10.1128/mBio.00886-19 (PMC6581854; doi:10.1128/mBio.00886-19)
Supplement: TEXT S1 [file mBio.00886-19-s0001.docx]

**Supplementary Information**

**The Lipid A 1-phosphatase, LpxE, Functionally Connects Multiple Layers of Bacterial Envelope Biogenesis**

**Running title: LpxE plays multiple roles in cell envelope biogenesis**

**Jinshi Zhao^a,1^, Jinsu An^b,c,1^, Dohyeon Hwang^b,c,1^**, **Qinglin Wu^a,1^, Su Wang^a^, Robert A. Gillespie^a^, Eun Gyeong Yang^b^, Ziqiang Guan^a^, Pei Zhou^a,^**^2^, **and Hak Suk Chung^b,c,^**^2^

^a^Duke University Medical Center, Department of Biochemistry, Durham, 27710, USA

^b^Center for Theragnosis, Biomedical Research Institute, Korea Institute of Science and Technology, Seoul 02792, Republic of Korea

^c^Division of Bio-Medical Science & Technology, KIST School, Korea University of Science and Technology, Seoul 02792, Republic of Korea

^1^These authors contributed equally: Jinshi Zhao, Jinsu An, Dohyeon Hwang, Qinglin Wu

^2^To whom correspondence should be addressed. Pei Zhou at 919 668-6409; Fax: 919 684-8885;peizhou@biochem.duke.edu; Hak Suk Chung at +82-(02) 958-6423; Fax +82-(02) 958-5805; hschung@kist.re.kr

**Supplementary Methods**

**Extraction and mass spectrometry analysis of lipid A from heptosyl-deficient strains expressing LpxE_AA_**

Either pWSK29 or pWSK29-LpxE_AA_ was expressed in the heptosyl-deficient strain WBB06 (1) at 37 °C. Cells were harvested at the OD_600_ of 1.0, and total lipids were extracted by the two-phase acidic Bligh-Dyer method (2). Dried lipids were re-suspended in 4:1 chloroform:methanol (v:v) and equal volumes of lipid samples were subjected to normal phase LC-MS analysis using an Agilent 1200 Quaternary LC system coupled with the QSTAR XL mass spectrometer as previously described (3). Data were analyzed using the Analyst QS software (Applied Biosystems).

**Confirmation of the lipid A 1-phosphatase activity of LpxE_AA_ from membrane extract**

C41(DE3) cells expressing LpxE_AA_ were grown in the ZYM-5052 auto-induction media (4). Harvested cells were lysed by a cell cracker (Microfluidics Corp.) in a lysis buffer containing 20 mM HEPES (pH 8.0), 300 mM NaCl, 2 mM EDTA, and Calbiochem protease inhibitor cocktail set II. Cellular debris was removed by centrifugation at 8,000 x *g* for 20 minutes. Membranes were pelleted at 150,000 x *g* for one hour and then re-suspended in 20 mM HEPES (pH 8.0) and 300 mM NaCl for assays. Kdo_2_-[4'-^32^P]-lipid IV_A_ was prepared from the disaccharide 1-phosphate precursor and [γ-^32^P]ATP as previously described (5). The lipid A 1-phosphatase reaction mixture consisted of 50 mM MES pH 6.0, 0.1% DDM, 0.5 mg/mL BSA, 10 µM Kdo_2_-lipid IV_A_, and 700 cpm/µL Kdo_2_-[4'-^32^P]-lipid IV_A_. Assays were carried out at 30 °C. After desired reaction times, reactions were quenched by spotting on a silica gel TLC plate. The TLC plate was developed in a solvent system of CHCl_3_:Pyridine:Formic Acid:H_2_O (30:70:16:10, v:v) and analyzed with a Phosphorimager.

**Cloning and purification of LpxE_AA_, LpxE_FN_, and UppP_FN_**

LpxE_AA_ was cloned into a modified pET26b vector as the C-terminal fusion to PelB-MBP separated by a linker peptide (LEVLFQGPAVPR) containing the PreScission protease cleave site. LpxE_AA_ was expressed in C41(DE3) cells using either the LB medium or SelenoMet^TM^ medium (Molecular Dimensions) and induced with 1 mM IPTG at 30 °C for 5 hours. After harvest, cells were lysed with French press in a buffer containing 20 mM HEPES (pH 8.0) and 300 mM NaCl, and LpxE were extracted from the cell lysate in the presence of 1% (v/w) DDM. After centrifugation at 20,000 rpm for 30 minutes, LpxE_AA_ from the supernatant was further purified with HisPur Cobalt affinity chromatography and the detergent was exchanged into 1% n-octyl-β-d-glucopyranoside (β-OG; Anatrace). The fusion protein was cleaved on column using the PreScission protease at 4 °C overnight, and the cleaved target protein was collected from the flow through, concentrated and purified through a Superdex200 size-exclusion chromatography in a buffer containing 25 mM Tris (pH 8.0), 150 mM NaCl, 2 mM tris(2-carboxyethyl)phosphine (TCEP), 0.8% β-OG. The peak samples were combined and concentrated for enzymatic and crystallization trials.

LpxE_FN_, and UppP_FN_ were cloned in modified pTRCHis vectors (ThermoFisher). LpxE_FN_ was expressed as an N-terminal His_10_-MBP fusion protein containing a C-terminal strep tag, and UppP_FN_ was expressed as an N-terminal His_10_-MBP fusion protein. Both proteins were expressed in C41(DE3) cells and induced with 1 mM IPTG for 5 hours. The proteins were purified using procedures similar to that used for the purification of LpxE_AA_, except that DDM was used throughout the purification. LpxE_FN_ was purified using the Ni^2+^-NTA and Strep-tacin columns, and UppP_FN_ was purified with the Co^2+^-NTA affinity column. The proteins were further purified through size-exclusion chromatography (Superdex 200, GE Healthcare) in a buffer containing 20 mM HEPES (pH 8.0), 300 mM NaCl, 1 mM DTT, 0.34 mM DDM for enzymatic assays.

Additional plasmids used in the genetic and cellular studies harboring various target genes are listed in Table S2. PCR templates for *lpxE_HP_* and *ftn_1552* were synthesized by IDT with a silence mutation at corresponding Ser17 (AGC to TCG in *lpxE_HP_*) and Ala203 (GCT to GCG in *ftn_1552*), respectively to remove a HindIII restriction site (AAGCTT). All constructs were verified by DNA sequencing.

***In vitro* enzymatic analysis of LpxE_AA_, LpxE_FN_, and UppP_FN_ with Kdo_2_-lipid A, C_55_-PP and PGP**

Kdo_2_-lipid A was purchased from Avanti Polar Lipids. The protocol for synthesis and purification of C_55_-PP was adapted from the method described previously for radiolabeled C_55_-PP substrate (6). The *E. coli* undecaprenyl pyrophosphate synthase (UppS) was similarly purified except using our expression vector pET16-*uppS_EC_*. The isopentenyl-pyrophosphate and farnesyl pyrophosphate were purchased from Sigma. The reaction volume was increased ten folds to 3 mL to obtain larger quantity and the radiolabeled isopentenyl-pyrophosphate was not used. The synthesis and purification of PGP followed the procedure described previously for Phosphatidyl[U-^14^C]Glycerol Phosphate with minor changes (3). The reaction volume was increased to 1.8 mL to yield a larger amount of final product and the [U-^14^C]glycerol 3-phosphate was replaced with cold glycerol 3-phosphate (Sigma) at 10 time excess over CDP-DAG (Avanti Polar Lipids). The *E. coli* phosphatidylglycerophosphate synthetase with C-terminal His10 tag was expressed from pET21-*pgsA_EC_* in the C41(DE3) strain. The cell culture was first incubated at 37 °C until density reached OD_600_ ~ 0.5, followed by overnight induction at 20 °C with 0.5 mM IPTG. Finally, the PgsA_EC_ was purified on Ni^2+^-NTA-agarose column in buffer containing 20mM HEPES (pH 8.0), 300mM NaCl and 0.34 mM of DDM.

To determine the specific activities of each enzyme with various substrates, a malachite green assay kit (Sigma) was used to monitor the release of free phosphate. All assays contained 100 μM substrates and were carried out at 37 °C. The enzyme amount and the time of reaction were adjusted so that enzyme activity is linear with time. The assay conditions were adapted from previous enzymatic studies for each substrate. With Kdo_2_-lipid A, the assay mixture consisted of 50 mM Tris-HCl (pH 7.5), 0.1% (w/v) Triton X-100 (7). With C_55_-PP, the assay mixture consisted of 50 mM Tris-HCl (pH 7.5), 0.1% (w/v) DDM and 150 mM NaCl (6). With PGP, the assay mixture consisted of 50 mM Tris-HCl (pH 7.5), 0.1% Triton X-100, 2 mM MgCl_2_ and 5 mM β-mercaptoethanol (3). The assays were quenched with 3.75 M formic acid and the yield of free phosphate concentration was determined by malachite green assay kit according to the product protocol.

To account for presence of the additional His tags and MBP fusion partners in the purified enzymes, the effective enzyme concentrations were calculated as the fraction of enzyme molecular weight over the total molecular weight including the additional tags and fusion partners. The reported specific activities were calculated with the effective enzyme concentrations.

**X-ray crystallography data collection and analysis**

The concentrated LpxE_AA_ sample (10.9 mg/mL) was mixed 1:1 (v:v) with mother liquor containing 90 mM HEPES (pH 7.0), 45 mM Li_2_SO_4_, 9% glycerol, 34.2% PEG600, 0.19 mM CYMAL-7. Crystals were obtained after incubation at 15 °C for ~ 2 weeks. The selenium single anomalous dispersion (Se-SAD) data set was collected at the wavelength of the selenium absorption peak (0.97911 Å) at the Southeast Regional Collaborative Access Team (SER-CAT) 22ID beamline, whereas the native dataset was collected at the Northeastern Collaborative Access Team (NECAT) 24IDC beamline at the Advanced Photon Source at Argonne National Laboratory. Diffraction data were processed with XDS and AIMLESS module in the CCP4 suite. Homology models of LpxE_AA_ were constructed using the templates of *E. coli* acid phosphatase (PDB: 1D2T) (8) and PgpB (PDB: 4PX7) (9), *Salmonella typhimurium* PhoN acid phosphatase (PDB: 2IPB) (10), and the vanadium-dependent iodoperoxidase (PDB: 4CIT) (11). Only the convergent segments of the models (residues P100-G168) were used as the search model. The phase information was obtained by using a combined molecular replacement and selenomethionine single-wavelength anomalous dispersion (MR-SAD) using the AutoSol module in the Phenix suite (12). A low resolution model was constructed using a 3.1 Å SAD dataset, which was used as the search model to phase a higher-resolution (2.38 Å) native dataset. Iterative model building and refinement were carried out using Coot (13) and Phenix (12), respectively. The coordinate has been deposited to RCSB (PDB ID: 6EBU).

**Construction of the C_55_-PP phosphatase deficient *E. coli* strain carrying the pMAK705-plasmid encoding target genes**

The *kan* cassette of Δ*ybjG::kan* in JW5112 (14) were removed using pCP20 (15) and the resulting strain was named as KHSC0007 (BW25113 Δ*ybjG*). A *pgpB::kan* cassette was introduced into KHSC0007 by P1 phage (16) generated from JW1270 (14) strain to yield KHSC0008 (BW25113 Δ*ybjG* Δ*pgpB::kan*). The *kan* cassette of *pgpB::kan* cassette was subsequently removed using pCP20 (15) to yield KHSC0009 (BW25113, Δ*ybjG,* Δ*pgpB*).

The temperature-sensitive plasmid pMAK705 encoding *bacA_EC_* (pMAK-*bacA_EC_*), *lpxE_AA_* (pMAK-*lpxE_AA_*), *lpxE_FN_* (pMAK-*lpxE_FN_*), *lpxE_HP_* (pMAK-*lpxE_HP_*), *lpxE_RL_* (pMAK-*lpxE_RL_*), or *uppP_FN_* (pMAK- *uppP_FN_*) was transformed into KHSC0009 by the TSS method (17). The *bacA::kan* cassette was introduced into these strains by P1 transduction (16). The names of the resulting strains are listed in Table S3. Deletions of *ybjG*, *pgpB*, and *bacA::kan* were confirmed by PCR.

**Construction of KAJS0005 (BW25113 Δ*ybjG*Δ*bacA*Δ*pgpB::P_L_-lpxE_AA_-FRT-kan-FRT*)**

A *bacA::kan* cassette was introduced into KHSC0007 by P1 phage (16) generated from JW3029 (14) strain and the *kan* cassette of *bacA::kan* cassette was subsequently removed using pCP20 (15) to yield KAJS0004 (BW25113 Δ*ybjG*Δ*bacA*).

In order to obtain the pgpB::*lpxEAA-FRT-kan-FRT* cassette*, lpxE_AA_* was inserted into a pBAD30 vector with XbaI and HindIII sites and then the kanamycin resistant cassette with two FRT sites, *FRT-kan-FRT* , was inserted into pBAD30-*lpxE_AA_* using HindIII recognition site. *araC* and P_BAD_ promoter were replaced with pL promoter by fused PCR using primer pairs and pBAD30-*lpxEAA-FRT-kan-FRT*. The resulting plasmid was named as pPLpro-*lpxE_AA_-FRT-kan-FRT*. For homologous recombination of *pgpB* on *E. coli* chromosome, PCR fragments, *P_L_-lpxE_AA_-FRT-kan-FRT* with pgpB up and downstream sequences, were amplified using primer pairs and pPLpro-*lpxE_AA_-FRT-kan-FRT* as a template. The *pgpB* gene was knocked out by *pgpB::P_L_-lpxE_AA_-FRT-kan-FRT* using DY330 strain (18). A *pgpB::P_L_-lpxE_AA_-FRT-kan-FRT* cassette was introduced into KAJS0004 by P1 phage (16) generated from KAJS0003 (14) strain to yield KAJS0005 (BW25113, Δ*ybjG*Δ*bacA*Δ *pgpB::P_L_-lpxE_AA_-FRT-kan-FRT*). Deletions of *ybjG*, *bacA*, and *pgpB* were confirmed by PCR.

**Growth dependency of *E. coli* C_55_-PP phosphatase deficient strains complemented by target genes**

C_55_-PP phosphatase deficient strains (BW25113, Δ*ybjG*Δ*pgpB*Δ*bacA::kan*) complemented by pMAK-*bacA_EC_*, pMAK-*lpxE_AA_*, pMAK-*lpxE_FN_*, pMAK-*uppP_FN_*, pMAK-*lpxE_RL_* or pMAK-*lpxE_HP_* along with the parental wild-type BW25113/pMAK705 strain were grown at 30 °C in the presence of 30 μg/mL chloramphenicol in LB. Bacterial cultures were diluted to an OD_600_ of 0.125; 5 μL of 10^2^-10^5^ diluted bacterial cultures were spotted on LB plates supplemented with 30 μg/mL chloramphenicol 30 °C or 42 °C for up to 2 days, respectively. The overexpression of BacA_EC_, LpxE_AA_, LpxE_FN_, and UppP_FN_ in the presence of 1 mM IPTG complements C_55_-PP phosphatase deficiency in *E. coli*, whereas leaky expression of LpxE_RL_, LpxE_HP_ or UppP_FN_ is sufficient to maintain bacterial viability at 30 °C.

**Construction of the PGP phosphatase deficient *E. coli* strain carrying the pMAK705-plasmid encoding target genes**

pMAK-*lpxE_AA_* was transformed into the Δ*pgpA*Δ*pgpB* double knockout mutant YL10 (W3110 Δ*pgpA*Δ*pgpB*) (3) by electroporation. A P1 phage generated from the strain JW5408 (14) was used to transduce the *pgpC*::*kan* cassette to the YL10/pMAK-*lpxE_AA_* strain to generate YL24/pMAK-*lpxE_AA_*. Transductants were selected at 30 °C on LB-agar plates containing 50 μg/mL kanamycin, 30 μg/mL chloramphenicol, 5 mM sodium citrate, and 0.3 mM IPTG. The deletion of *pgpA, pgpB*, and *pgpC::kan* were confirmed by PCR. While we were successful in obtaining the PGP phosphatase deficient strain complemented by pMAK-*lpxE_AA_*, we failed to achieve complementation by pMAK-*lpxE_FN_.*

**Construction of KAJS0009 (W3110 Δ*pgpA* Δ*pgpB::P_L_-lpxE_AA_*Δ*pgpC::kan*)**

A *pgpB::P_L_-lpxE_AA_-FRT-kan-FRT* cassette was introduced into KAJS0007 by P1 phage (16) generated from KAJS003 (14) strain and the *kan* cassette of *pgpB::P_L_-lpxE_AA_-FRT-kan-FRT* cassette was subsequently removed using pCP20 (15) to yield KAJS0008 (W3110 Δ*pgpA*Δ*pgpB::P_L_-lpxE*). *pgpC* gene was knocked out using the *pgpC::kan* cassette following the procedures described above. Deletions of *pgpA*, *pgpC,* and *pgpB* were confirmed by PCR.

**Growth dependency of PGP phosphatase deficient *E. coli* strain complemented by *lpxE_AA_***

The PGP phosphatase deficient strain (W3110, Δ*pgpA*Δ*pgpB*Δ*pgpC::kan*) complemented by *pgpA_EC_* (YL24/pMAK-*pgpA_EC_*) (16) or *lpxE_AA_* (YL24/pMAK705-*lpxE_AA_*) were tested using the similar procedures as described for C_55_-PP deficient strains with 0.3 mM IPTG.

**Growth phenotypes of KAJS0005 and KAJS0009**

KAJS0005 and KAJS0009 were grown in LB medium containing 50 μg/mL of kanamycin and wild type *E. coli* (BW25113 and W3110), KAJS0004, and KAJS0008 were grown in LB medium at 37 °C. 10 μL overnight cultures of each strain were spotted and streaked on LB agar plates.

**Constructions of *F. novicida* U112 strains with genetic deletions**

The chromosomal deletion mutants (JZ1801-1803 and JZ1805, Table S3) in *Fransicella novicida* were constructed by the spontaneous homologous recombination method (19). For mutants JZ1803 and JZ1805, linear DNA fragments of the FRT-site containing kanamycin resistance cassette T20 (20) flanked by ~2 kb upstream and downstream regions of the gene of interest were chemically transformed to *F. novicida* (21). The kanamycin resistant colonies were selected and the gene deletion was confirmed by PCR and sequencing. In the case of JZ1801 and JZ1802, as well as XWK1, similar protocol applied except that no FRT-site was included in the insertion. For JZ1801 and JZ1802, a tetracycline resistance cassette was used to disrupt the *lpcC* gene.

In order to construct JZ1807, the *kan* cassette was first removed from JZ1805 by transforming the tetracycline-resistant plasmid pLG72 (20), which carries the FLP recombinase. After the removal of the *kan* cassette was confirmed by PCR and kanamycin sensitivity test, the strain spontaneously cured of pLG72 (JZ1806) was isolated after growth in Tryptic Soy Broth supplemented with 0.1% cysteine (TSB-C) medium without antibiotic selection. The chromosomal replacement of *lpxE_FN_* with *lpxE_AA_* was achieved as described above by transforming into JZ1806 using a linear DNA fragment of the synthetic *pelB*-*lpxE_AA_* gene and FRT-site containing kanamycin resistance cassette T20 (20) flanked by ~1.5 kb upstream and downstream regions of the *lpxE_FN_* gene.

To construct double gene deletion mutant JZ1808, the kanamycin cassette in JZ1803 was similarly removed to yield JZ1804. Due to the synthetic lethality of *lpxE* and *uppP* double mutation, plasmid pEDL17-*lpxE_FN_* was first introduced before the second round of mutagenesis to delete the *uppP_FN_* gene to yield JZ1808 (U112, Δ*lpxE*Δ*uppP* (*ftn_1552*)*:: FRT-kan-FRT* /pEDL17-*lpxE_FN_*).

Plasmid pEDL17-*lpxE_FN_* is a TetR-based gene expression system in *F. novicida* with tightly controlled expression level of LpxE_FN_. To construct the plasmid, the coding sequence of LpxE_FN_ was cloned in pEDL17 (22), an anhydrotetracycline-inducible expression plasmid, using the in-fusion cloning kit (Takara) replacing its mOrange2 coding sequence behind the promoter. The plasmid was confirmed by sequencing.

**Characterization of *F. novicida* mutants**

*Growth phenotype*: To study the growth phenotype of strain JZ1808 (U112 Δ*lpxE*Δ*uppP::FRT-kan-FRT*/pEDL17-*lpxE_FN_*) with and without aTc, the strain was first grown in the TSB-C medium supplemented with 250 ng/mL aTc overnight at 37 °C in a shaking incubator. The wild-type (WT) *F. novicida* U112 strain was also grown in parallel in the TSB-C medium as a control. The overnight cultures were diluted 1000 folds into fresh TSB-C medium with or without 250 ng/mL aTc and incubated for 16 hours at 37 °C. The absorption at 600 nm (OD_600_) of the cultures was determined, and the cells were diluted to OD_600_ about 0.02 in fresh pre-warmed TSB-C medium with or without 250 ng/mL aTc. The cultures were incubated at 37 °C and the OD_600_ was monitored every 30 minutes. To maintain log phase growth, the culture was diluted 10x in a fresh medium once the OD_600_ reading reached or passed 0.2.

*Lipid profile*: The total lipids were extracted from *F. novicida* strains by the Bligh-Dyer extraction method. The lipids were dissolved in chloroform: methanol (4:1, v/v) and applied to a TLC plate. After development in a solvent system with chloroform: methanol: pyridine: acetic acid: water (25:10:5:4:3, v/v), the lipids were visualized by spraying 10% sulfuric acid in ethanol and charred on a hot plate. To analyze the new lipid species accumulated in *F. novicida* mutants, the preparative TLC was employed as described previously (7). After analyzing the extracted lipids with ESI/MS with direct injection, the predominant peaks were matched to expected masses of different lipid species.

*Susceptibility to bacitracin*: The susceptibility of the *F. novicida* wild-type (WT), Δ*lpxE::kan* and Δ*uppP::kan* strains to bacitracin was determined measurements of the minimum inhibitory concentration (MIC). The MIC assay protocol was adapted from methods described in National Committee for Clinical Laboratory Standards (NCCLS) to using 96-well plates. Bacteria were grown in the TSB-C medium at 37 °C in the presence of varying concentrations of bacitracin. To obtain more accurate readings of the MICs, three series of twofold dilutions of inhibitors were used. The starting concentrations of the three series are different by factors of 1.33 and 1.67, respectively. MICs were reported as the lowest compound concentration that inhibited bacterial growth.

*Cell morphology:* To observe *F. novicida* strains (WT and Δ*lpxE*Δ*uppP*::*kan*) under the microscope, the overnight culture in TSB-C medium without anhydrotetracycline (aTc) was first diluted to OD_600_ = 0.1 and 2 μL of the diluted culture was spotted on a thin TSB-C agar pad on a glass slide and covered with a glass coverslip. The *F. novicida* Δ*lpxE*Δ*uppP*::*kan* strain grown in the presence of 200 ng/mL aTc was also included for comparison. The slide was observed under the Zeiss Axio Observer live cell station (Light Microscopy Core Facility at Duke University) using 100x oil objective lens, and differential interference contrast images were recorded.

*Change of O-antigen repeats of LPS*: In order to monitor the change of O-antigen repeats of LPS, overnight cultures of *F. novicida* strains without aTc were diluted to OD_600_ = 1 in 1 mL TSB-C and the cells were harvested and re-suspended in 50 μL of water. An equal volume of 2x Laemmli buffer was added, and the samples were frozen at -80 °C for 10 minutes followed by boiling for 10 minutes. After another cycle of freezing-and-boiling, the samples were cooled and proteinase K was added to a final concentration of 0.2 mg/mL. The samples were incubated for 3 hours at 55 °C and were used for electrophoresis analysis on AnyKD SDS-PAGE gel (Bio-Rad). The gel was stained with Pro-Q Emerald 300 Lipopolysaccharide Gel Stain Kit (Molecular Probes) and visualized using a UV transilluminator.

**References:**

1. Brabetz W, Muller-Loennies S, Holst O, & Brade H (1997) Deletion of the heptosyltransferase genes *rfaC* and *rfaF* in *Escherichia coli* K-12 results in an Re-type lipopolysaccharide with a high degree of 2-aminoethanol phosphate substitution. *Eur J Biochem* 247(2):716-724.

2. Bligh EG & Dyer WJ (1959) A rapid method of total lipid extraction and purification. *Can J Biochem Physiol* 37(8):911-917.

3. Lu YH, Guan Z, Zhao J, & Raetz CR (2011) Three phosphatidylglycerol-phosphate phosphatases in the inner membrane of *Escherichia coli*. *J Biol Chem* 286(7):5506-5518.

4. Studier FW (2005) Protein production by auto-induction in high-density shaking cultures. *Protein Expression and Purification* 41(1):207-234.

5. Karbarz MJ, Six DA, & Raetz CR (2009) Purification and characterization of the lipid A 1-phosphatase LpxE of Rhizobium leguminosarum. *J Biol Chem* 284(1):414-425.

6. Manat G*, et al.* (2015) Membrane Topology and Biochemical Characterization of the *Escherichia coli* BacA Undecaprenyl-Pyrophosphate Phosphatase. *PLoS One* 10(11):e0142870.

7. Wang X, McGrath SC, Cotter RJ, & Raetz CR (2006) Expression cloning and periplasmic orientation of the Francisella novicida lipid A 4'-phosphatase LpxF. *J Biol Chem* 281(14):9321-9330.

8. Ishikawa K, Mihara Y, Gondoh K, Suzuki E, & Asano Y (2000) X-ray structures of a novel acid phosphatase from Escherichia blattae and its complex with the transition-state analog molybdate. *EMBO J* 19(11):2412-2423.

9. Fan J, Jiang D, Zhao Y, Liu J, & Zhang XC (2014) Crystal structure of lipid phosphatase Escherichia coli phosphatidylglycerophosphate phosphatase B. *Proc Natl Acad Sci U S A* 111(21):7636-7640.

10. Makde RD, Mahajan SK, & Kumar V (2007) Structure and mutational analysis of the PhoN protein of Salmonella typhimurium provide insight into mechanistic details. *Biochemistry* 46(8):2079-2090.

11. Fournier JB*, et al.* (2014) The Vanadium Iodoperoxidase from the Marine Flavobacteriaceae Species Zobellia galactanivorans Reveals Novel Molecular and Evolutionary Features of Halide Specificity in the Vanadium Haloperoxidase Enzyme Family. *Applied and Environmental Microbiology* 80(24):7561-7573.

12. Adams PD*, et al.* (2010) PHENIX: a comprehensive Python-based system for macromolecular structure solution. *Acta Crystallogr D Biol Crystallogr* 66(Pt 2):213-221.

13. Emsley P & Cowtan K (2004) Coot: model-building tools for molecular graphics. *Acta Crystallogr D Biol Crystallogr* 60(Pt 12 Pt 1):2126-2132.

14. Baba T*, et al.* (2006) Construction of *Escherichia coli* K-12 in-frame, single-gene knockout mutants: the Keio collection. *Mol Syst Biol* 2:2006 0008.

15. Datsenko KA & Wanner BL (2000) One-step inactivation of chromosomal genes in *Escherichia coli* K-12 using PCR products. *Proc Natl Acad Sci U S A* 97(12):6640-6645.

16. Miller JH (1972) Experiments in Molecular Genetics. *Cold Spring Harbor Laboratory Press*.

17. Chung CT, Niemela SL, & Miller RH (1989) One-step preparation of competent *Escherichia coli*: transformation and storage of bacterial cells in the same solution. *Proc Natl Acad Sci U S A* 86(7):2172-2175.

18. Yu D*, et al.* (2000) An efficient recombination system for chromosome engineering in Escherichia coli. *Proc Natl Acad Sci U S A* 97(11):5978-5983.

19. Llewellyn AC*, et al.* (2012) NaxD is a deacetylase required for lipid A modification and *Francisella* pathogenesis. *Mol Microbiol* 86(3):611-627.

20. Gallagher LA*, et al.* (2007) A comprehensive transposon mutant library of *Francisella novicida*, a bioweapon surrogate. *Proc Natl Acad Sci U S A* 104(3):1009-1014.

21. Gallagher LA, McKevitt M, Ramage ER, & Manoil C (2008) Genetic dissection of the Francisella novicida restriction barrier. *J Bacteriol* 190(23):7830-7837.

22. LoVullo ED, Miller CN, Pavelka MS, Jr., & Kawula TH (2012) TetR-based gene regulation systems for *Francisella tularensis*. *Appl Environ Microbiol* 78(19):6883-6889.

23. Sievers F*, et al.* (2011) Fast, scalable generation of high-quality protein multiple sequence alignments using Clustal Omega. *Molecular Systems Biology* 7.

24. Tong SL*, et al.* (2016) Structural Insight into Substrate Selection and Catalysis of Lipid Phosphate Phosphatase PgpB in the Cell Membrane. *Journal of Biological Chemistry* 291(35):18342-18352.
